# Functional Enrichment and Sequence-Based Discovery Identify Promiscuous and Efficient Poly Lactic Acid Degrading Enzymes

**DOI:** 10.1021/acs.est.4c07279

**Published:** 2025-04-01

**Authors:** Gorjan Stojanovski, Maria Bawn, Amy Locks, Esther Ambrose-Dempster, John M. Ward, Jack W. E. Jeffries, Helen C. Hailes

**Affiliations:** † Department of Biochemical Engineering, University College London, Bernard Katz Building, London WC1E 6BT, U.K.; ‡ Department of Chemistry, University College London, 20 Gordon Street, London WC1H 0AJ, U.K.

**Keywords:** polymer degradation, plastics, enzyme discovery, peptides and proteins, genome mining, compostable plastic

## Abstract

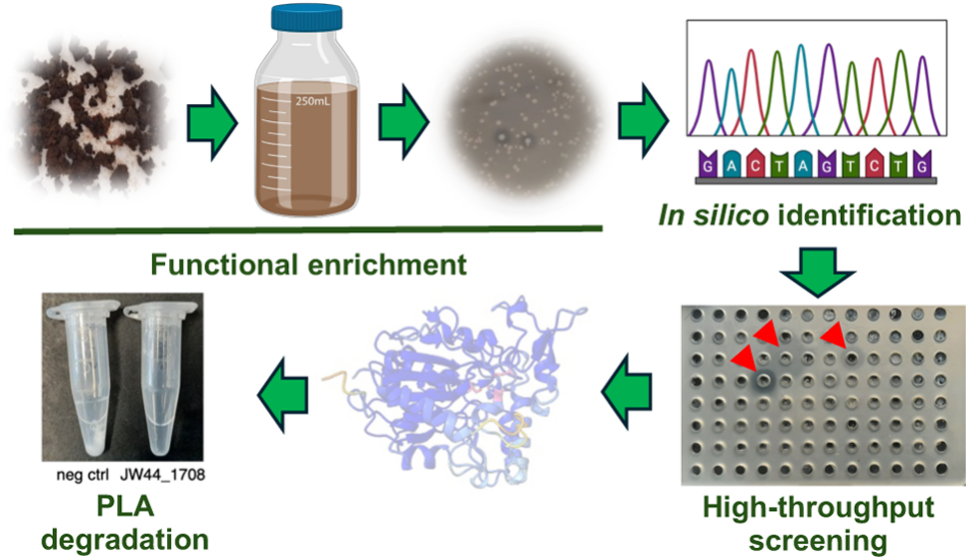

The recalcitrance of petroleum-based plastics to recycling has prompted the use of alternative compostable materials such as poly lactic acid (PLA) and polybutylene terephthalate coadipate (PBAT). However, current preferred end-of-life waste management solutions, such as aerobic composting and anaerobic digestion, are not optimal for bioplastics, due to their slow and variable degradation rates. Thus, the isolation of novel microbes and their plastic-degrading enzymes is necessary to improve existing bioplastic disposal and create more sustainable routes to valorize waste plastic. In this study, through functional enrichment cultures, we isolated 14 unique microbes capable of PLA and PBAT degradation and applied a computational discovery pipeline to identify plastic-degrading enzymes. Through this, a focused set of 97 enzymes was functionally characterized, finding three active PLA-degrading enzymes. The two most active enzymes, JW45_1534 and JW44_1708, displayed broad polyester degrading activity against PLA, PBAT, PBSA, PCL, and Impranil polyurethane. Uniquely, under optimized reaction conditions, JW44_1708 fully solubilized low-molecular-weight PLA powder (100−500 mM lactic acid equivalents) in 18 h at 30 °C, with 43−65% conversion to monomeric lactic acid. Overall, we demonstrate the effectiveness of functional enrichment with single-pass computational filtering and screening for finding highly active PLA-degrading enzymes with the potential to improve PLA end-of-life waste management solutions.

## Abstract 2

This study reports the isolation and characterization of novel PLA- and PBAT-degrading enzymes using functional enrichment cultures and an *in silico* pipeline for plastic-degrading enzyme discovery. Notably, one enzyme found was able to degrade PLA powder in 18 h at 30 °C, with up to 65% of lactic acid formed and the remaining powder fully solubilized as PLA oligomers.

## Introduction

Global plastic production has reached 359 million tonnes per annum.^1^ However, only 9% of plastic is recycled, with the majority going to landfill (49%), being incinerated (19%), or leaking into the natural environment (22%).^2^ In 2024, bioplastics accounted for 2 million tonnes of total global plastic production, which is set to increase to 5 million tonnes by 2029.^3^ Biobased and biodegradable polymers such as poly lactic acid (PLA) have been proposed as potential alternatives to petroleum-based plastics particularly for single-use applications. However, while appealing as a solution, bioplastics still face the same issues as the petroleum-based plastics they are intended to replace, such as unsuitable end-of-life recycling infrastructure, slow and differing biodegradation rates in different environments,^4^ and long-term environmental persistence.

Preferred end-of-life strategies to degrade PLA and other bioplastics include industrial aerobic biodegradation under thermophilic (50–60 °C) composting conditions and anaerobic digestion, which can typically take several months.^5−8^ Unfortunately, these degradation rates are still slower than the typical anaerobic digestion time scale of 15−30 days for municipal food waste.^5^ If degradation rates of PLA could be enhanced, then it could be disposed of alongside organic waste in industrial composting and anaerobic digestion facilities without the need for separation.^6^ Identifying microbes that can degrade bioplastics and enriching aerobic or anaerobic streams with these organisms could be one route to increase the degradation efficiency of bioplastics in waste disposal processes. Alternatively, developments in plastic separation technologies such as hyperspectral imaging,^7,8^ which could be used at waste plants, would allow these plastics to be separated from mixed waste streams. These bioplastics could then be degraded in an enzymatic process to recover the monomer units, either for a closed loop process back to virgin biopolymer or used as feedstocks for other value-added products.^9^ Thus, identifying both plastic-degrading enzymes and the microbes from which they are derived is necessary to improve existing bioplastic recycling processes.

PLA is degraded by serine hydrolase enzymes, which catalyze either end cleavage (“exo” cleavage) of the polymer releasing monomeric lactic acid or generate oligomeric products via internal cleavage of polymer chains (‘endo’ cleavage). Polyesterase activity is functionally poorly defined and appears to be a promiscuous activity of several hydrolytic enzyme classes such as lipases, esterases, and proteases.^10−12^ While notable examples of PLA-degrading enzymes exhibiting high PLA degradation rates (30−60 mM release of monomeric lactic acid in 18 h of reaction) have been reported,^10,13,14^ many other natural enzymes isolated still show quite slow degradation rates.^11,15,16^ Due to the poor functional classification, identifying highly active enzymes from genomic databases is challenging, as the search space is vast. As the preferred end-of-life options for bioplastics are industrial composting and anaerobic digestion, we aimed to use a combination of functional and computational identification methods of plastic-degrading enzymes (PDEs) to significantly reduce our search space for active bioplastic degrading enzymes.

The key aim of this study was, therefore, to discover natural bioplastic degrading enzymes that have the potential to increase the efficiency of PLA degradation. This would then enable applications as isolated enzymes or ultimately their use in the microbes from which they are derived in the open environment or in anaerobic digestors. Through functional enrichment cultures, 14 unique microbes were isolated from industrial and home composting soils capable of PLA and PBAT degradation. Using a general *in silico* and experimental platform for plastic-degrading enzyme discovery, three enzymes capable of PLA degradation were identified from these microbes. Characterization and reaction engineering resulted in an enzyme capable of tolerating high PLA substrate concentrations (0.5 M) and the full solubilization of solid PLA powder in less than 1 day.

## Results and Discussion

**Figure 1:**
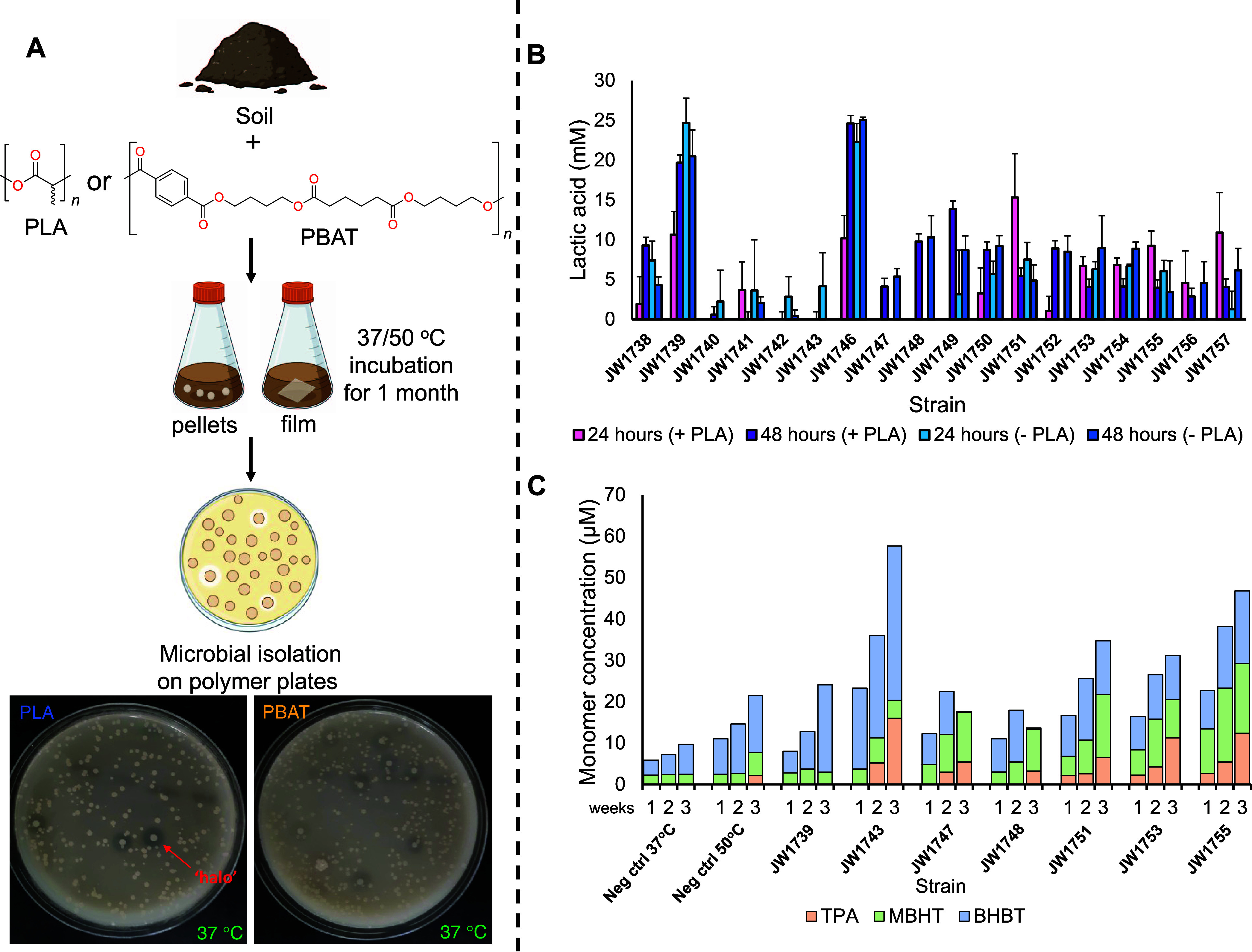
Functional enrichment identified 14 novel plastic-degrading microbes. (A) Overview of the functional enrichment and microbial isolation strategy used. Seven soil samples (Table S1) were coincubated with high-molecular weight PLA and PBAT pellets or film in liquid growth media for 1 month at either 37 or 50 °C. After incubation, microbial isolates were screened for PLA- and PBAT-degrading activity on emulsified agar plates.^21^ Representative plates for PLA and PBAT degradation are shown at the bottom, where clear zones (‘haloes’) around colonies that degrade either plastic are highlighted. (B) PLA powder degradation and assimilation. Strains were incubated with PLA powder (pink and purple bars) and without PLA powder (blue bars) in growth media at their respective growth temperature (Table 1) for 48 h. Samples of the broth were taken after 24 and 48 h, and L-lactic acid production was quantified by HPLC. Bars represent the means of triplicate cultures and error bars represent the standard deviation. Inactive strains are not plotted. (C) PBAT film degradation. Strains were coincubated with a PBAT film for 3 weeks, and samples were taken after 1, 2, and 3 weeks of incubation. Monomer release was quantified by HPLC using an external product standard. Bars represent single measurements, and inactive strains are not plotted.

Novel plastic-degrading enzymes (PDEs) have been isolated directly from wild-type micro-organisms and metagenomic DNA or improved by protein engineering.^9,13,14,17−19^ However, identifying natural micro-organisms that produce PDEs is advantageous as both the enzymes identified, and their native microbes can be used for plastic waste valorization. In this respect, functional enrichment by coincubating isolated soil/marine samples in the presence of plastic in otherwise nutrient-poor media conditions has been particularly effective in identifying naturally occurring plastic-degrading organisms.^20−22^ Thus, we applied this approach to both identify novel microbes and reduce the sequence search space to identify and characterize the PDEs responsible for plastic degradation. Seven soil samples from home compost and industrial composting sites (Table S1) were incubated with PLA and PBAT plastics to identify micro-organisms capable of plastic degradation (Figure 1A). Samples were incubated under mesophilic (37 °C) and thermophilic (50 °C) temperatures as they represent conditions present in industrial aerobic composting sites and in anaerobic digestors (35−55 °C), which are preferred recycling solutions for compostable plastics. Zone-clearing assays identified 23 organisms capable of plastic degradation, 16 of which could degrade PLA, and 7 of which could degrade PBAT.

Interestingly, 60% (14/23) of the strains were isolated from soil from an industrial aerobic composting site, potentially due to the longer-term adaptation of soil organisms to higher levels of plastic contamination compared to conditions in other soils (Tables 1 and S2).

Whole-genome sequencing indicated that 83% (19/23) were Bacilli with 14 unique isolates. Notably, no soil pretreatment was performed to preferentially isolate certain microbes, suggesting that Bacilli may outcompete and represent primary plastic degraders in naive soil samples. One strain, *Brevibacillus agri* was separately isolated 6 times from 3 different soil samples possibly suggesting the wider prevalence of this strain in soil compared to other isolated microbes. Through this approach, novel microbes were both identified, and the total possible sequence search space for responsible PDEs was reduced (to 108,000 genes) compared to naive *in silico* genomic database mining and with lower labor compared to raw functional metagenome screening. The isolated strains were then coincubated with both low-molecular-weight PLA powder and Ecoflex PBAT film for 2 days and 3 weeks, respectively, and monomer release was characterized (Figure 1B,1C). To account for lactic acid production from microbial fermentation, all strains were also incubated without PLA powder to determine background levels of lactic acid produced by the strains over the 2-day incubation. Over the course of incubation with PLA powder, the strains released from 1 to 25 mM of lactic acid; however, this was comparable to the negative controls which lacked PLA powder (Figure 1B), indicating that most of the lactic acid came from microbial fermentation. In fact, across both time points, only JW1738, JW1749, JW1751, JW1752, JW1755, JW1756, and JW1757 showed more lactic acid production (0.4−5 mM) when incubated with PLA powder compared to controls without PLA powder (Figure 1B). Additionally, JW1751, JW1755, JW1756, and JW1757 showed decreased levels of lactic acid at 48 h compared to 24 h of incubation, potentially indicating some assimilation of lactic acid by the strains for metabolism. Conversely, PBAT degradation products accumulated over time, showing a mix of PBAT monomers—terephthalic acid (TPA), monobutylene-terephthalate (MHBT), and bis-butylene terephthalate (BHBT), with the TPA fraction increasing over the course of incubation (Figure 1C). Two strains degraded both plastics (JW1751 and JW1755), with five degrading PLA only (JW1738, JW1749, JW1752, JW1756, and JW1757) and one degrading PBAT only (JW1743). Overall, PBAT monomer release was quite low (5−60 μM) and the vast majority of PLA produced by the strains came from microbial fermentation in these experiments. However, this is not unusual as conditions that stimulate PDE expression in natural organisms are often not known, and PDE expression can vary depending on screening conditions. As all strains were shown to produce clear zones on initial plates that contained PLA/PBAT plastic, all strains likely still contained potential PDEs that could be identified.

**Table 1:** Table of Isolated Strains and Identities

strain	enrichment substrate	soil sample	enrichment temperature (°C)	organism (% identity)
JW1738	PLA	Hotbin starter	37	*Lysinibacillus sphaericus* DSM 28 (100%)
JW1740	PLA	Teabag compost	50	*Hydrogenophaga temperata* strain TR7-01 (97.94%)
JW1741	PLA	Hotbin starter	50	*Aneurinibacillus thermoaerophilus* DSM 10154 (96.57%)
JW1742	PLA	Industrial compost	50	*Aeribacillus pallidus* DSM 3670 (97.11%)
JW1744	PLA	Teabag compost	50	*Bacillus licheniformis* DSM 13 (99.81%)
JW1745	PLA	Industrial leachate	50	*Caldibacillus hisashii* N-11 (99.48%)
JW1746	PLA	Industrial compost	37	*Brevibacillus agri* DSM 6348 (99.92%)
JW1749	PLA	Industrial leachate	37	*Brevibacillus agri* DSM 6348 (99.92%)
JW1750	PLA	Hotbin starter	37	*Bordetella trematum* DSM 11334 (97.11%)
JW1752	PLA	Industrial leachate	37	*Rummeliibacillus pycnus* NBRC 101231 (100%)
JW1754	PLA	Industrial compost	37	*Bacillus sanguinis* BML-BC004 (99.87%)
JW1756	PLA	Industrial compost	37	*Bacillus sanguinis* BML-BC004 (96.59%)
JW1757	PLA	Teabag compost	37	*Brevibacillus agri* DSM 6348 (99.93%)
JW1758^a^	PLA	Industrial compost	50	*Bacillus licheniformis* DSM 13 (100%)/*Aneurinibacillus thermoaerophilus* DSM 10154 (99.87%)
JW1759	PLA	PET hotbin	50	*Bacillus paralicheniformis* KJ-16 (100%)
JW1739^a^	PBAT	Teabag compost	37	*Brucella intermedia* LMG 3301 (99.69%)/*Shigella sonnei* CECT 4887 (93.63%)
JW1743	PBAT	Industrial compost	50	*Brevibacillus borstelensis* DSM 6347 (100%)
JW1747	PBAT	Industrial leachate	37	*Brevibacillus agri* DSM 6348 (99.53%)
JW1748	PBAT	Teabag compost	37	*Lysinibacillus fusiformis* DSM 2898 (99.01%)
JW1751	PBAT	Industrial compost	37	*Bacillus sanguinis* BML-BC004 (99.87%)
JW1753	PBAT	Industrial leachate	37	*Brevibacillus agri* DSM 6348 (97.18%)
JW1755	PBAT	Industrial compost	37	*Brevibacillus agri* DSM 6348 (99.93%)
JW1760	Impranil	Impranil emulsion plate		*Bacillus licheniformis* DSM 13 (100%)

a = For these strains, two 16S rRNA genes were found by barrnap suggesting mixed cultures of two bacteria.

### Conservative *In Silico* Genome Mining Identifies a Focused Set of Plastic-Degrading Enzymes (PDEs) for Functional Assessment

**Figure 2:**
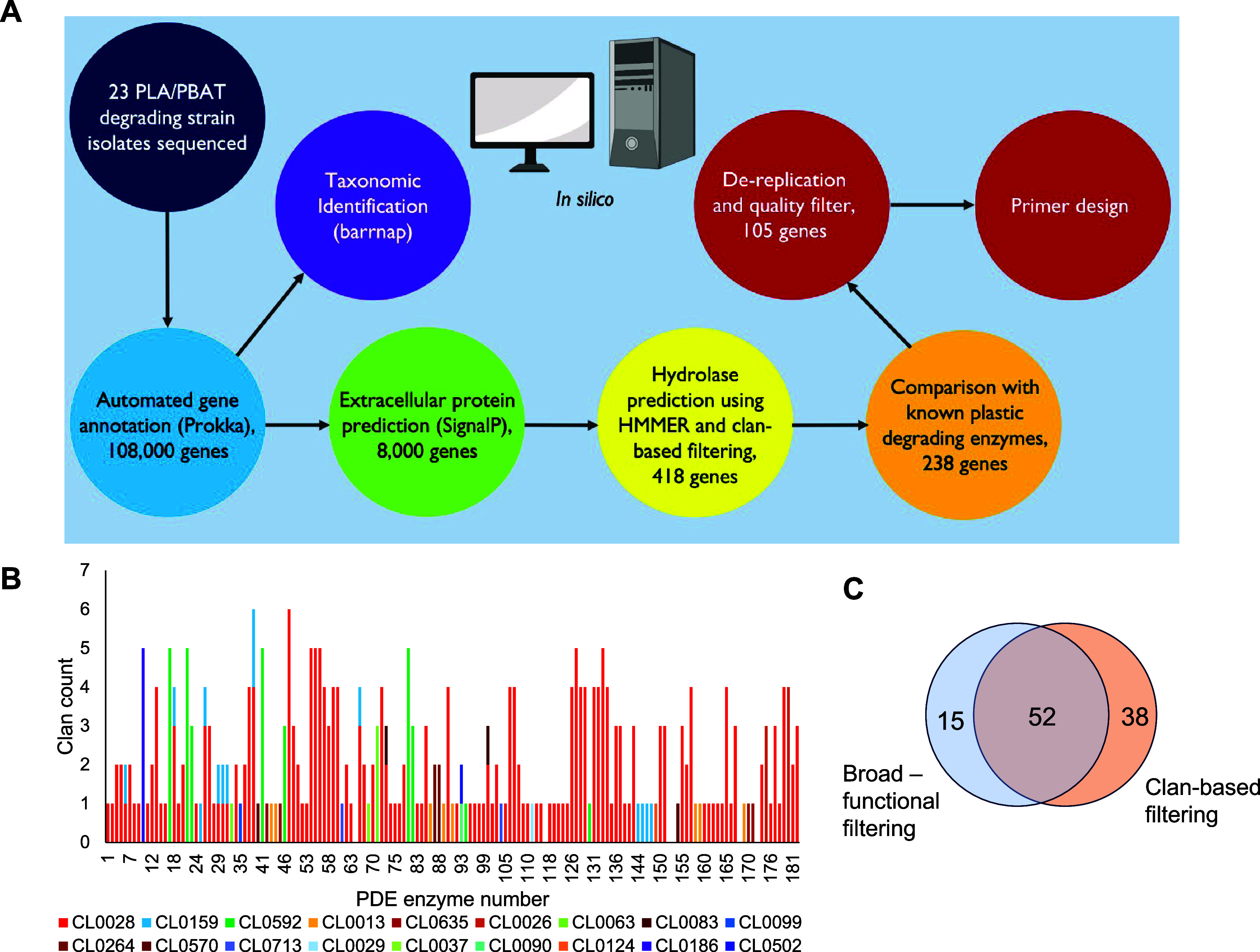
General functional prediction pipeline to generate focused libraries of PDEs. (A) Overview of the pipeline developed, and the number of remaining enzymes present after each stage of analysis using the second strategy described for the 23 isolated organisms. (B) Bar chart showing the functional annotations present in 182 plastic-degrading enzymes from the PlasticDB.^23^ The quantification of the number of occurrences of each clan annotation among the 182 PDEs is presented in Table S4. (C) Overlap of genes was obtained from the two functional filtering approaches.

To identify PDEs responsible for degradation from the isolated microbes, a computational pipeline was designed and applied (Figure 2A). Prediction of secretion peptide signals (Table S3) was used as an initial filter to focus exclusively on extracellularly secreted enzymes, as all natural PDEs need to be secreted to act on the polymeric substrate.

Secretion peptide sequences are also present in 86% (157/182) of known PDEs in the PlasticDB as identified by SignalP,^23,24^ validating this as an appropriate initial filtering method. To classify PET degrading enzymes, Danso and co-workers applied an iterative cycle of computational prediction and experimental validation to confidently identify natural enzymes capable of PET degradation.^24^ Iterative cycles were required as the biodegradation of man-made polymers is a rare and evolutionarily novel catalytic activity. It is also a side activity of several different hydrolytic enzyme classes such as lipases, proteases, hydrolases, and esterases.^10,25−28^ This makes single-pass functional filtering a challenge, as well-defined and robust models for functional identification are not currently available. As such, we applied a two-prong conservative classification approach. Initially, broad functional annotations (protease, hydrolase, lipase, esterase, and cutinase) were generated and then compared with annotations of known PLA/PBAT-degrading PDEs from the PlasticDB database.^23^ This approach produced 254 sequences which, after removal of duplicate sequences, yielded 67 potential PDEs. However, one drawback of this approach is that enzymes that contain only accessory domains present in known PLA/PBAT-degrading enzymes but lack catalytic domains can also be recovered, which can complicate downstream functional screening.

Therefore, a second approach was applied that focused on identifying enzyme catalytic domains. Catalytically related protein families have been grouped into “clans” enabling broad classification of several protein families that may contain PDEs.^29^ As such, known PDEs in the PlasticDB were analyzed for clan annotations, and 18 unique clans were found, with PLA/PBAT-degrading enzymes being present in clans CL0028, CL0013, CL0124, CL0264, and CL0570 (Figure 2B, Table S4). Using the clan annotations for the known enzymes, 418 sequences were obtained, which were further filtered using family specific functional annotations for known PDEs (Table S5), reducing the number of sequences to 238. After quality control filters and removal of highly similar sequences (see Materials and Methods for details), a list of 90 potential PDEs was obtained. Comparison of the sequences obtained from both approaches showed a high degree of overlap (Figure 2C) and to maximize sequence coverage for functional testing, the sequences from both approaches were combined giving a final set of 105 enzymes for functional characterization (Table S6).

### Functional Screening of Putative PLA/PBAT-Degrading Enzymes

To facilitate efficient functional characterization of the putative PDEs, a high-throughput molecular biology screening platform was utilized by using an automated liquid handling robot (Figure S1). Applying this platform, gene sequences were amplified, cloned, transformed, and expressed with excellent (75−90%) first-pass success rates (Table S7). Overall, 97 enzymes from the original 105 were successfully obtained for functional characterization, with the remainder failing due to challenges in PCR amplification and cloning. For functional characterization, the resulting enzymes were tested in agar well-clear-zone assays against emulsified PLA and PBAT polymers. Initial characterization showed putative zones of clearing in every well. This was ascribed to the CHAPS lysis reagent used (Figure S2), and switching to alternative cell lysis agents ameliorated this issue. Only one enzyme was identified from this initial screen. Heterologous protein production can be a significant challenge when characterizing novel enzymes, thus the protein expression conditions were optimized to account for rare codons present in the sequences (Figure S3) and increase cell growth (Table S8), and subsequent clear-zone assays indicated 2−3 enzymes which degraded PLA. Further inspection also suggested 12−13 enzymes with minor zones of clearing (Table S9). Two enzymes JW1745_1534 and JW1744_1708 showed consistent zones of clearing across multiple rounds, while the remaining 13 enzymes were subjected to secondary confirmation screens using crudely purified enzymes. Under these conditions, only one additional enzyme (JW1751_1026) showed zones of clearing against emulsified PLA (Figure S4). Overall, three enzymes were found from 97 total enzymes screened, showing a 3% success rate. While quite low, this is comparable to other PLA-degrading enzyme screening campaigns,^14,30^ highlighting the currently weak functional classification of these enzymes, necessitating conservative enzyme screening sets. Danso et al., utilized an iterative HMM model screening approach to identify putative PETase enzymes *in silico*.^24^ Notably applying a naive ‘PLAase’ HMM model (generated by sequence alignment of known PLA-degrading enzymes from the PlasticDB), while identifying JW1745_1534 and JW1744_1708, did not identify JW1751_1026 as a ‘PLAase’ enzyme (Table S9). This finding further highlights that the polyesterase activity is poorly functionally defined among hydrolytic enzymes. It also suggests that a conservative broad functional classification search strategy as utilized here can be effective in finding a greater number of PLA-degrading enzymes, particularly for single-pass screening and functional characterization.

The three active PLA-degrading enzymes (JW44_1708, JW45_1534, and JW51_1026) were isolated from *Bacillus licheniformis*, *Caldibacillus hisashii*, and *Bacillus sanguinis*, respectively (Table 1). Several previous studies have also isolated *Bacillus licheniformis* strains, which have shown degradative activity toward PLA, and several proteases and lipases have been isolated, which show polyesterase activity, potentially highlighting the wide distribution of this bacterium and its importance for degrading plastics in natural environments.^10,15,16,31^ To the best of our knowledge, no plastic-degrading enzymes have been reported from *Caldibacillus hisashii* and *Bacillus sanguinis*, possibly suggesting that these microbes may be unique to the soil environments we sampled. To determine similarity of the enzymes to previously characterized PLA and PBAT-degrading enzymes, the three enzymes were compared to 14 PBAT-degrading enzymes and 33 PLA-degrading enzymes previously reported^23^ (Figure S5). JW44_1708 displayed 58% sequence identity to a PLA-degrading enzyme from *Paenibacillus amylolyticus*(17) and 68% sequence identity to PBATH_Bp_ from *Bacillus pumilis*,^32^ JW45_1534 displayed ∼50% sequence identity to plaM4, plaM5, Cbotu_EstA, Cbotu_EstB, and PfL1,^15,26,33^ while JW1751_1026 had very low homology to known PLA and PBAT hydrolases (8−30%). Therefore, the three enzymes display a low similarity to previously characterized PLA/PBAT-degrading enzymes.

### Biochemical Characterization of PLA-Degrading Enzymes

**Figure 3:**
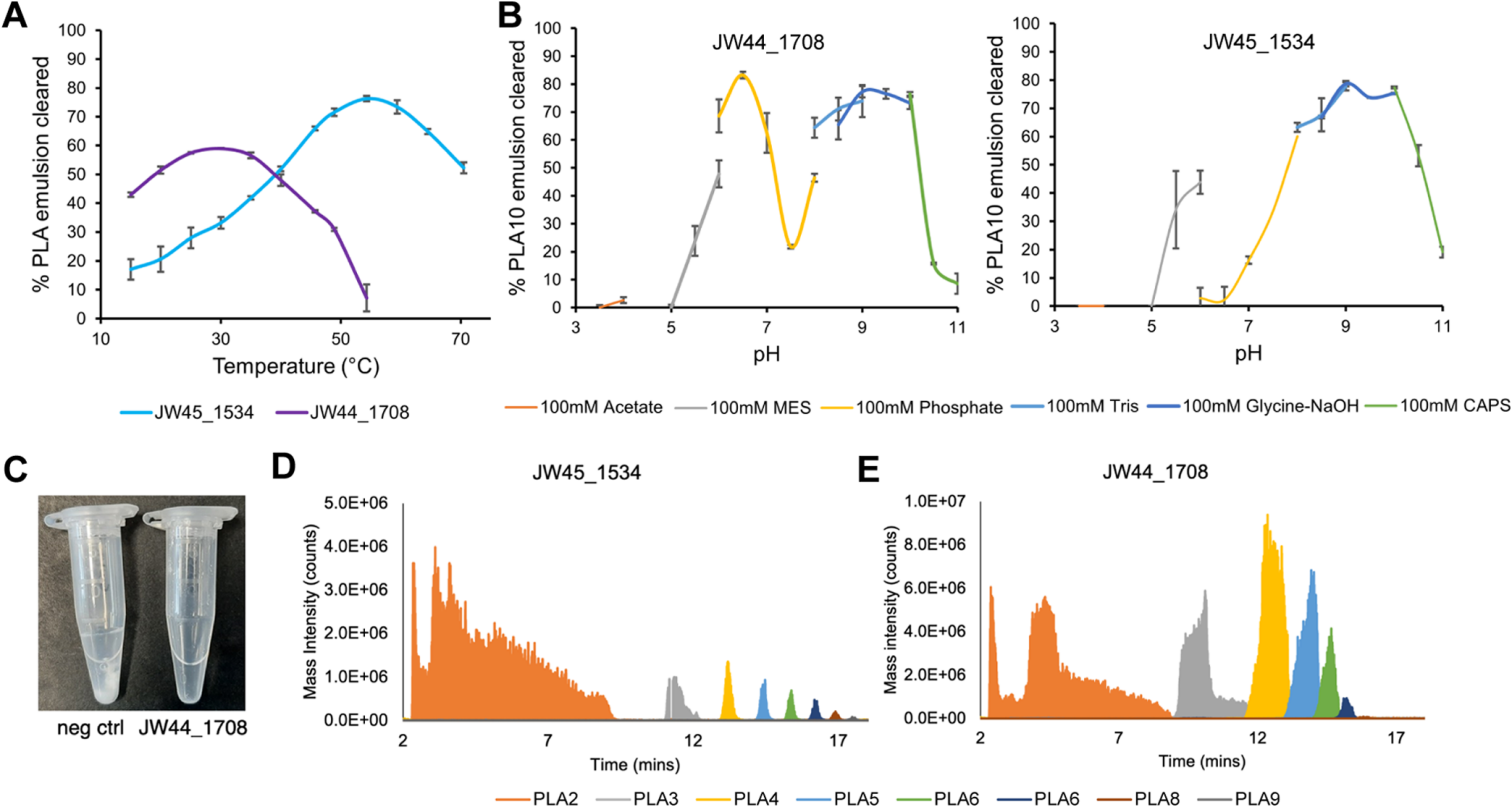
Biochemical characterization of JW44_1708 and JW1745_1534. (A) Temperature optimum determination. 0.25 mg/mL of each purified enzyme was incubated with 20% v/v of a low-molecular-weight PLA (*M*_w_ ∼ 10,000) emulsion in 50 mM phosphate buffer pH 8.0 for 1.5 h at 15−70 °C. After incubation, absorbance was measured at 580 nm. % PLA emulsion cleared was determined by comparison with the absorbance of a no enzyme control incubated under the same conditions. Lines are the means of triplicate experiments and error bars represent the standard deviation (<±5%). (B) pH optimum screening. pH optima were determined using the same turbidimetric approach as for temperature optimum determination, reactions were incubated for 2 h at 37 °C in different buffers all at 100 mM and absorbance was measured every 90 s. After 30 min of incubation, the turbidity reduction was quantified and compared relative to a no enzyme control. Lines are the means of triplicate experiments and error bars represent the standard deviation (<±6%). (C) Image comparing the remaining solid powder after 22 h of reaction with 0.25 mg/mL of JW44_1708 enzyme. While solid powder is still present in the no enzyme control, the powder is fully solubilized in the JW44_1708 reaction. (D) Mass spectrometry analysis of the soluble fractions after 22 h reaction of 15 mg/mL PLA powder with JW1745_1534. An extracted ion chromatogram was obtained for each oligomer and then combined to give a composite figure. Traces are representative of triplicate measurements. PLA2, PLA3, PLA4,··· = PLA dimer, PLA trimer, PLA tetramer, .... (E) Mass spectrometry analysis of the soluble fractions after 22 h reaction of 15 mg/mL PLA powder with JW1744_1708. Figure is presented the same as for (D).

The two most active PLA-degrading enzymes identified, JW45_1534 and JW44_1708, were then further characterized to determine their optimal degradation conditions and substrate scope. The host strains JW1745 and JW1744 were isolated from industrial compost and a domestic hotbin, respectively, but JW1745 was isolated from a thermophilic (50 °C) PLA + soil enrichment while JW1744 was isolated from a mesophilic (37 °C) PLA + soil enrichment. This is also reflected in the optimal temperature of the enzymes, and JW45_1534 had the highest PLA degradation activity at 55 °C while JW44_1708 had an optimum of 30 °C (Figure 3A). Broad pH ranges were observed for both enzymes, with JW45_1534 showing activity from pH 7−10.5, while JW44_1708 was similarly active from pH 7−10 (Figure 3B). Clear optima were observed around pH 9.0 for both enzymes, similar to other known PLA-degrading enzymes.^13,14^ Many characterized PLA-degrading enzymes have broad polyester degrading activity, able to degrade polycaprolactone (PCL), polybutylene succinate adipate (PBSA), polybutylene succinate (PBS), polyethylene succinate (PES), poly(3-hydroxybutyrate co-3-hydroxyvalerate) (PHBV), and 3PET.^13,14^

Industrially, an enzyme capable of broad polyesterase activity is desirable, as such enzymes and the originating microbes can be used to degrade mixed plastic waste streams. As such, the two enzymes were screened against a panel of emulsified polyester plastics, which consisted of PLA of increasing molecular weights, polybutylene adipate coterephthalate (PBAT), PCL, PBSA, polyhydroxybutyric acid (PHBH), and Impranil polyurethane (PU). Interestingly, both enzymes degraded all of the emulsified polymers tested, demonstrating that both enzymes have broad polyester degrading activity (Figure S6).

### Reaction Optimization Enables Full Solubilization of PLA by JW44_1708

**Figure 4:**
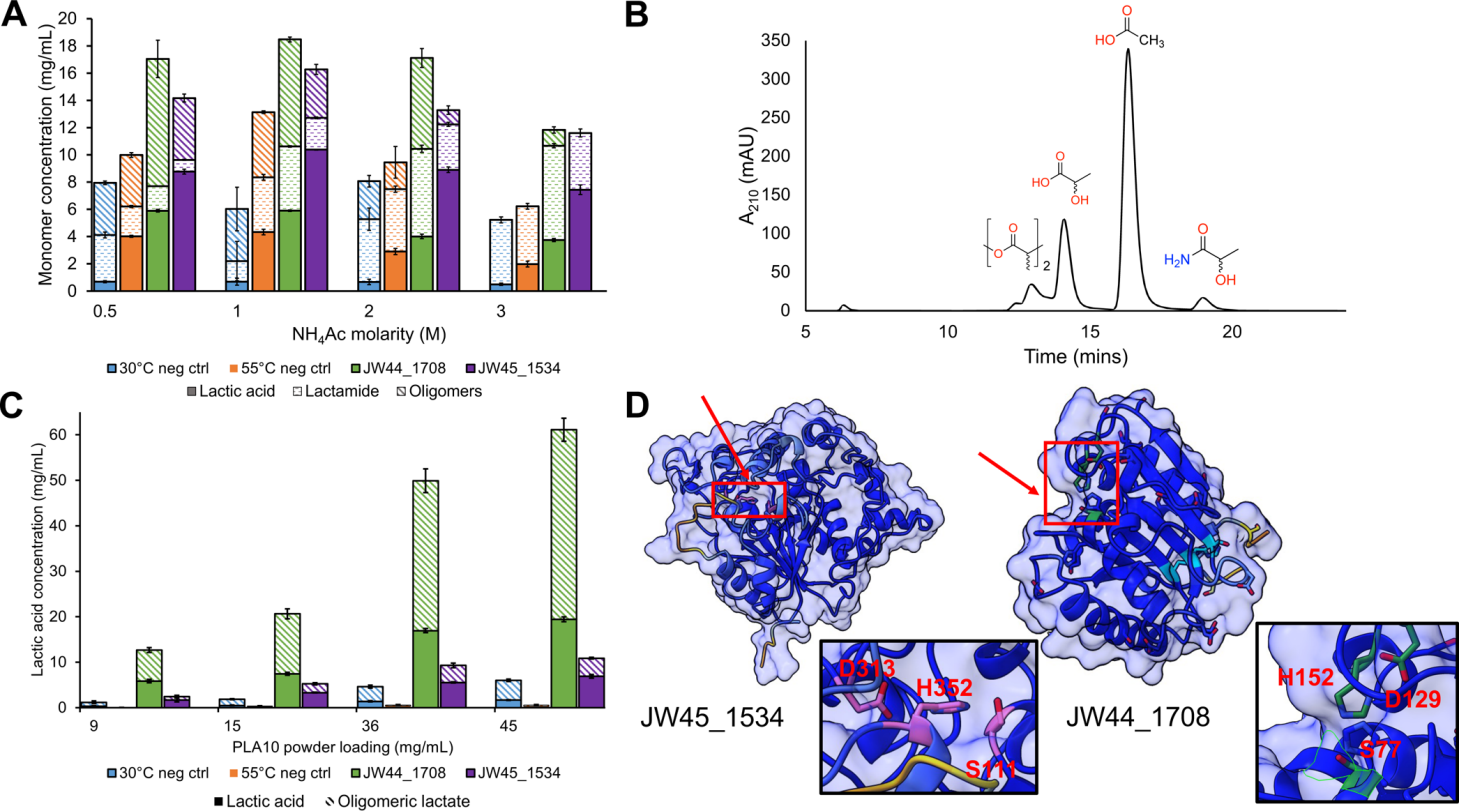
Reaction optimization allows a high-concentration product release by JW44_1708. (A) Buffer strength screening. 0.1 mg/mL of each enzyme was incubated with 15 mg/mL of PLA10 powder (1.5% *w/v*) in ammonium acetate pH 9.0 (0.5−3 M) for 18 h at either 30 °C (JW44_1708) or 55 °C (JW45_1534). Monomer concentration was quantified by HPLC (Figure 4B) using an external product standard. Oligomeric lactate concentration was determined following previously reported methods.^13^ The negative controls are the same reactions incubated at 30 and 55 °C without the enzyme. Bars are the averages of triplicate experiments and error bars represent the standard deviation (<±1.6 mg/mL). (B) Representative HPLC trace showing the elution profile of lactate oligomers, lactic acid, acetic acid (from the ammonium acetate buffer), and the lactamide side product. (C) Substrate loading assay. 0.1 mg/mL of each enzyme were incubated with increasing concentrations of PLA10 powder (9–45 mg/mL, 0.9–4.5% *w/v*) in 1 M Tris-HCl pH 9.0 for 18 h at either 30 °C (JW44_1708) or 55 °C (JW45_1534). Monomer concentration was quantified as described for the buffer strength assay. The negative controls are the same reactions incubated at 30 and 55 °C without enzyme. Bars are the averages of triplicate experiments and error bars represent the standard deviation (< ± 2.5 mg/mL). (D) Structural predictions suggest that JW44_1708 has a surface-exposed active site. Predicted structures were generated using ColabFold^36^ and visualized in Chimera X. Surfaces are colored using pLDDT. Active site catalytic triads are colored pink (JW45_1534 structure) and dark green (JW44_1708) and are highlighted by red arrows. Insets show the active site for each protein with the putative catalytic residues labeled.

With the two enzymes clearly showing activity against emulsified PLA, it was next tested whether they could degrade the solid PLA powder as a suspension. Racemic PLA powder (*M*_w_ ∼ 10,000) was incubated with purified JW45_1534 and JW44_1708 at 55 and 30 °C, respectively. Under these conditions, JW44_1708 showed complete solubilization of the PLA powder after overnight incubation, while some solid residues were still present in the JW45_1534 reaction (Figures 3C and S7). Mass spectrometry analysis showed that both enzymes were capable of ‘endo’ cleavage of PLA as PLA oligomers were observed for both enzymes, but the oligomeric profiles differed (Figure 3D). JW45_1534 mostly produced PLA dimers, and each higher-order oligomer decreased in intensity. Conversely, JW44_1708 produced dimer, trimer, and tetrameric PLA in high concentrations. This suggests that JW44_1708 may preferentially degrade longer chain substrates compared to shorter-chain substrates, while JW45_1534 may preferentially act on shorter-chain substrates. Notably the lactic acid monomer was not observed by mass spectrometry but was observed by HPLC, and was the predominant product formed by both enzymes (Figure 4A). Both enzymes were also tested against solid poly-l-lactic acid film; however, HPLC analysis did not show considerable lactic acid release even after 48 h of reaction. This may be due to a combination of decreased surface area and increased crystallinity of the film compared to the powder (Figure S8) and lower activity of the enzymes for pure L-lactic acid polymers. Current enzymatic recycling of plastic requires substantial pretreatment of the plastic both to reduce substrate crystallinity and increase the surface area of the polymer through micronization such that degradation occurs in efficient and economical time scales.^9,34^ Furthermore, particle size reduction of plastics by cryomilling is feasible for large-scale implementation. Therefore, these enzymes represent an excellent starting point for future studies to explore film degradation together with potential pretreatments to optimize PLA degradation.

Reaction acidification is a notable problem in polyester degradation, particularly when degradation releases soluble monomeric acids. The decrease in the reaction pH can lead to premature inactivation of the enzyme despite its capacity to tolerate greater product concentrations. This is typically ameliorated by using high buffer concentrations, however this can lead to side product formation due to buffer nucleophilicity, thus reducing recovery of the desired product, as observed for a cutinase from *Humicola insolens* during mechanoenzymatic degradation with high PLA substrate concentrations.^35^ In initial solid PLA degradation tests performed, for both enzymes, the pH dropped to 6−7 after overnight incubation. To test if increased buffer concentration was beneficial, solid PLA powder was degraded with increasing (0.5−3 M) buffer concentration (Figure 4A). It was observed that higher buffer concentrations of 0.5−1 M ammonium acetate did lead to increased yields of lactic acid; however, beyond 1 M concentration of buffer, product conversions decreased. Similarly, lactamide side product formation was observed which also increased as buffer concentration increased (Figure 4A and 4B). Lactamide formation was observed due to the nucleophilicity of free ammonia used in the buffer, and thus a less nucleophilic but strong buffering agent was necessary if higher substrate concentrations were to be used compared to the 15 mg/mL used initially. Tris buffer was selected as it showed very strong buffering capacity, and the bulky substituents made it less nucleophilic. Furthermore, it has been previously shown to lead to only minor (<4%) side product formation.^35^ Additionally, in the buffer strength screen applied (Figure 4A), considerable background nonenzymatic hydrolysis was observed particularly at 55 °C. This was due to pretreatment of the powder by sonication to generate a suspension. However, as this was not necessary for the enzymatic degradation, this pretreatment was omitted in subsequent experiments.

With an optimal buffer determined, the substrate loading was increased to 45 mg/mL (∼500 mM lactic acid equivalents) and incubated with the enzymes at their respective temperature optima in 1 M Tris pH 9.0 as this was sufficient to prevent reaction acidification and limit side product formation (Figure 4C). To account for the strong temperature dependence of Tris buffers, buffer solutions were heated to the optimal reaction temperature for each enzyme and then the pH was adjusted to 8.0 prior to use in assays. Under these conditions, JW44_1708 was observed to fully solubilize the PLA powder even at 45 mg/mL (4.5% *w/v*) of PLA while the residual solid PLA powder increased with increasing substrate concentration with JW45_1534 (Figure S7). With no pretreatment, background chemical hydrolysis was limited to 9% of total lactic acid/lactate oligomer release. When reaction acidification is prevented, JW44_1708 at ambient reaction temperature (30 °C) could release from 5.9 − 19.4 mg/mL of monomeric lactate (65−215 mM), corresponding to 43−65% conversion to monomeric product, with the remaining powder being solubilized as PLA oligomers. Conversely, JW45_1534 released 1.7−6.9 mg/mL of lactic acid (15−19% conversion to monomeric product), with lower (0.7−4 mg/mL) oligomeric product being released (Figure 4C). Thus, for JW45_1534, around 64% of the total product is monomeric lactate, while for JW44_1708, around 34% is monomeric lactate, again suggesting that JW45_1534 may preferentially degrade shorter-chain PLA oligomers compared to JW44_1708, which preferentially degrades longer chain PLA oligomers.

Structural predictions of each protein provide a rationale for this difference in polymer degradation (Figure 4D, Figure S9). While both display typical α/β hydrolase folds, JW45_1534s active site (consisting of Ser111-His352-Asp313) is predicted to be more buried in the core of the protein, while conversely, JW44_1708 has a more surface-exposed active site (consisting of Ser77-His152-Asp129) (Figure 4D, Figure S9). As such, the greater access to the JW44_1708 active site may permit preferential hydrolysis of the longer PLA chains and better interaction with the polymer surfaces. Conversely for JW45_1534, shorter-chain PLA oligomers are preferred, as they have easier access to the more buried active site compared to longer polymeric chains.

Notably, the predicted structure of JW44_1708 suggested the presence of a second potential catalytic triad (consisting of Ser120-His98-Asp72) in the β-sheet of the monomeric subunit with a similarly surface-exposed active site groove (Figure S10A). However, whether this was a catalytically functional site was yet to be determined. Notably, the wild-type JW44_1708 appeared to display two pH optima ranging from pH 5.5−7.5 and pH 7.5−10.5 (Figure 3B), which may be suggestive of two active sites. To test this, catalytic serine mutants S77A and S120A were generated and tested for their PLA emulsion-clearing activity at varying pH values. The S77A mutant displayed no PLA-degrading activity (in the basic pH range), thus confirming this as a catalytically active serine residue; however, some PLA de-emulsification was observed at lower pH values (Figure S10B). The S120A mutant showed no activity at lower pH values but normal PLA degradation at basic pHs similar to that of the wild-type enzyme (Figure S10B). HPLC analysis of the cleared wells for the WT enzyme showed only some lactic acid formation at basic pHs, while none was observed at acidic pHs (Figure S10C). Furthermore, covalent labeling of the WT enzyme with PMSF and mass spectrometry analysis indicated only single addition of a PMSF molecule, supporting the presence of a single active site in JW44_1708 (Figure S10D). Thus, while it is possible that the second putative active site may only be capable of forming higher-order PLA oligomers, which would account for the precipitation of the PLA emulsion at low pH in the presence of JW44_1708, overall, the data aligns with a single active site, displaying PLA degradation capability in the alkaline pH range. The de-emulsification observed at low pHs may be artifactual due to decreased emulsion stability in the presence of protein, which may reduce PLA turbidity. We have observed that PLA emulsions are unstable in certain buffer conditions (low pH acetate and citrate buffers around 4 − 6), and indeed, the larger errors and observation of some precipitation at lower pHs supports the conclusion of low pH de-emulsification as being an experimental artifact. However, JW44_1708 may represent an interesting starting point to artificially engineer a second protein active site in the monomeric polypeptide for applications in non-natural environments.

In summary, we utilized one month functional enrichment cultures to isolate 23 PLA/PBAT-degrading organisms, 14 of which were unique strain isolates. Genomic sequencing and computational selection using a broad functional classification approach guided by pre-existing PDE databases allowed the generation of a small set of 105 enzymes for functional screening. From this approach, three enzymes were found to display PLA-degrading activity in the emulsion plates. Prior studies have used iterative rounds of *in silico* screening (with HMM models) and functional testing to find metagenomic PETase enzymes.^24^ In our study, one of the PLA-degrading enzymes found did not match HMM models made from known PLA, PBAT, or PET degrading enzymes. This demonstrates the benefit of our broad functional classification approach for single-round functional screening, as active enzymes can be found that HMM models miss. This also highlights the current weak functional classification of PDEs, which is likely to improve as more enzymes are isolated and characterized.

Interestingly, neither JW1744 nor JW1745 displayed significant monomer release when tested as whole strains against PLA powder or PBAT film, yet highly active enzymes were found in these organisms. This highlights the variable expression of PDEs in wild-type organisms and the challenges of finding optimal conditions that permit plastic degradation. This was also observed for a PLA-degrading *Bacillus pumilis* strain, which varied widely depending on the carbon and nitrogen sources present in the media.^37^ This again shows the benefit of taking a conservative and combined functional/sequence-based approach, as highly active enzymes could be missed through functional screening alone.

Two of the enzymes were biochemically characterized and displayed similar pH optima to known PLA-degrading enzymes.^14^ Notably, prior studies used soluble para-nitrophenol esters as probe substrates for enzymatic characterization.^13,14,28^ However, the enzymatic activity on these substrates does not accurately reflect the polymer degradation activity. To account for this, we opted to fully characterize enzymes against polymer emulsions to directly assess the activity against PLA, thereby directly analyzing polyesterase activity. Reaction acidification has been previously shown to limit reaction yields in PLA degradation assays due to the release of lactic acid.^35^ Through optimizing reaction conditions, JW44_1708 was shown to be capable of fully solubilizing solid PLA powder with 42 − 65% lactic acid formation. While comparison of enzymes between studies is challenging due to the variability of polymer substrates used, our study used similar reaction conditions and identical PLA powder substrates to two other studies, which reported highly active PLA-degrading enzymes (Table 2). For the industrial studies, a different PLA substrate was used.^19^ Notably, the monomer release of JW44_1708 is similar to other highly active metagenomic PLA depolymerases, ABO2449, MGS0156, and GEN0105^13,14,30^ (Table 2), suggesting that JW44_1708 is a promising candidate enzyme for ambient temperature enzymatic PLA recycling. Additionally, although a putative second active site was not shown to be catalytically active, the enzyme scaffold is an attractive target to engineer a novel depolymerase with two catalytic sites within a single monomer unit.

**Table 2:** Comparison of Lactic Acid Release from PLA Powder using PLA-Degrading Enzymes

enzyme	mg_enzyme_/g_PLA_	temperature (°C)	lactic acid yield (%)^a^	reference
JW45_1534	6.7	55	22	
	2.2		15	this study
JW44_1708	6.7	30	50	
	2.2		42	this study
GEN0105	4.2	30	36	(14)
MGS0156	4.2	30	45	(14)
MGS0156 S169A	4.2	30	30	(14)
ABO2449	4.2	36	40	(13)
RPA1511	4.2	36	12	(13)
PAM	0.15	45	96	(19)
Proteinase K	0.15	45	18	(19)
Thc_cut2	4.2	30	12	(14)
PlaM4	4.2	36	3	(13)

a Lactic acid conversion was compared after 18 h of reaction. Reaction conversion after 24 h was compared for PAM and Proteinase K, and a different PLA substrate was used in our study.

Recently, Carbios reported embedding an engineered PLA depolymerase for programmed degradation of PLA-based yogurt pots post-consumption. For this application, a hyper-thermostable PLA depolymerase was engineered with a melting temperature of 80 °C, which could withstand the polymer extrusion process.^19^ The high degradation rates observed with JW44_1708 in this study under high substrate loading (4.5% w/v PLA) and the initial thermostability of JW45_1534, which had an optimum temperature of 55 °C, make both enzymes promising candidates to engineer thermostable PLA depolymerases, which could similarly be embedded into PLA plastics as a way of enhancing PLA degradation under composting conditions. Additionally, engineering these enzymes for thermostability can also harness the synergistic benefit of using ionic liquids at elevated temperatures to degrade PLA.^38^ Furthermore, the two host strains identified in this study, *Bacillus licheniformis* strain JW1744 and *Caldibacillus hisashii* strain JW1745 and other microbes isolated, could be used directly to enhance microbial degradation of PLA in open environment settings such as industrial composting and anaerobic digestion. Further characterization of conditions to improve natural PDE expression and microbial depolymerization will enable realization of their degradative potential.

## Materials and Methods

### Preparation of Bioplastic Soil Enrichment Cultures and Isolation of Plastic-Degrading Microbes

Sources of reagents, plastic samples, and compost samples are detailed in Extended Methods and Table S1. One gram of each compost sample was suspended in 20 mL of nutrient broth no. 2 (1/4 recommended mass) (Oxoid), containing 1 g of high-molecular-weight PLA granules (Goodfellow, ME34-GL-000110) or PBAT (Ecoworld Biodegradable Polymer) pellets and were incubated. Following incubation, samples were taken and spread onto agar plates containing nutrient broth (1/4 recommended mass) and 10% (v/v) of emulsified PLA or PBAT, and plates were incubated for 1 week at 37 or 50 °C. The colonies forming clear zones indicating plastic emulsion degradation were selected for genomic DNA isolation and whole-genome sequencing.

### *In silico* Bioinformatic Analysis of Putative Plastic-Degrading Enzymes (PDEs)

To identify putative PDEs, an *in-silico* analysis pipeline was developed (Figure 2A). Closely related organisms to those isolated were determined using *barrnap*(39) by identification of the 16S rRNA gene and BLAST analysis against the NCBI database. Annotated genes were initially analyzed by SignalP 6.0^40^ to predict which enzymes contained N-terminal secretion peptides (Table S3). This showed that per genome, 5−10% of all proteins were predicted to be secreted, and this subset was focused on for PDE discovery. To predict putative PDEs, two conservative approaches were taken. The first approach filtered the resulting 8080 sequences from SignalP 6.0 analysis using *HMMER v3*.3.2^41^ with the default settings to obtain functional predictions for each sequence. These sequences were then filtered for sequences that were predicted to be hydrolases, lipases, esterases, proteases, and cutinases. This was then further filtered by comparison with a public database of PDEs (PlasticDB^23^). The 182 sequence PlasticDB database was also analyzed by *HMMER v3*.3.2 to determine functional annotations that occur in experimentally validated PDEs. The resulting list of functional annotations was used to more stringently filter the predicted sequences. The sequences obtained were then dereplicated by sequence identity using a percentage identity matrix generated using *ClustalOmega*(42) and a sequence identity threshold of >90%. This first approach yielded 67 putative PDE sequences.

A second approach was also taken, as it was observed that *HMMER v3*.3.2 annotated only 6015 sequences, leaving 2065 sequences unannotated. To address this, the 8080 sequences were analyzed using *pfamscan*(42) with a permissive E-value threshold of 10. This increased the number of annotated sequences to 7232 sequences, reducing the number of unannotated sequences to 848 sequences. The remaining unannotated sequences were then analyzed using *interproscan*(43) to obtain annotations from other databases. To ensure all sequences contained catalytic domains relevant for PDE degradation, the PlasticDB^23^ was analyzed by *pfamscan* to determine the distribution of clan annotations. The predicted sequences were then filtered based on clans which contained enzymes that were experimentally shown to degrade PLA or PBAT—CL0013, CL0028, CL0124, CL0264, CL0570, and S8_Peptidases. This approach yielded 418 sequences, which were then further filtered using the family specific sequence annotations for experimentally characterized PDEs from the PlasticDB. This yielded 238 sequences. To ensure sequence diversity, the sequences were dereplicated by sequence identity as for the first approach, yielding 127 sequences. To address the permissive *E*-value threshold of *pfamscan*, enzymes were excluded if the E-value of the annotation was >0.01 and if the amino acid length of the sequence was greater than 1000 amino acids (the longest experimentally characterized PDE in the PlasticDB is 734 aa). This gave a final set of 90 enzymes. The annotated enzymes from both approaches were compared, finding 52 shared enzymes, 15 enzymes exclusive to the first approach and 38 enzymes exclusive to the second. The enzymes from both approaches were combined to give a final test set of 105 putative PDEs (Table S6).

### High-Throughput Screening of Plastic-Degrading Enzymes

Clarified cell lysates of putative plastic degraders were tested for PLA emulsion-clearing activity in an agar-plate clearing assay. The agar test plates contained 2% agar +50 mM potassium phosphate buffer pH 8.0 and 10% (v/v) emulsified PLA. Wells were punched into the agar plates with a custom-built 96-well plate puncher, and 30 μL of clarified lysate was loaded into each well. Plates were incubated at 37 °C for 48 h to assess enzymatic emulsion-clearing activity.

### Biochemical Characterization of JW44_1708 and JW45_1534

#### Temperature Assay

The temperature optimum was determined using a turbidimetric emulsion-clearing assay similar to previous reports.^14^ Reaction conditions were as follows; 50 mM potassium phosphate buffer pH 8.0, 20% (v/v) low-molecular-weight Resomer(R) 202-H PLA emulsion (PLA10), and 0.25 mg/mL enzyme. The reactions were setup in triplicate, with a control lacking enzyme setup in duplicate in PCR tubes. Reactions were incubated for 1.5 h at 15−70 °C in 5 °C increments in a ProFlex PCR thermocycler (Applied Biosystems). Following incubation, reaction mixtures were transferred to a 96-well plate, and the optical density was measured at 580 nm using a plate reader (CLARIOstar Plus Microplate Reader, BMG LabTech). Absorbance values were averaged across replicates and compared relative to the no enzyme control to quantify the extent of plastic emulsion-clearing.

#### pH Assay

Enzymatic pH optimum was determined by using a turbidimetric emulsion-clearing assay. Reaction conditions were as follows; 100 mM buffer, 20% (v/v) low-molecular-weight Resomer(R) 202-H PLA emulsion (PLA10), and 0.25 mg/mL enzyme. The following buffers were used to cover different pH ranges: Sodium acetate pH 3.5−4.0, MES pH 5.0−6.0, potassium phosphate pH 6.0−8.0, Tris pH 8.0−9.0, Glycine-NaOH pH 8.5−10, CAPS pH 10−11. The reactions and control lacking enzyme were setup in triplicate, in a 96-well plate. Reactions were incubated for 2 h at 37 °C/300 rpm in a plate reader (CLARIOstar Plus Microplate Reader, BMG LabTech), monitoring absorbance at 580 nm every 90 s. Absorbance values after 30 min of incubation were averaged across replicates and compared relative to the no enzyme control to quantify the extent of plastic emulsion-clearing.

#### Polyesterase Emulsion-Clearing Assays

To determine the promiscuity of polyesterase activity of the PLA-degrading enzymes, emulsion-clearing assays were prepared with different polymers. Ecoflex PBAT, PBSA, PCL, PHBH, and PLA of varying molecular weight (PLA10, PLA55, PLA107, PLA148, and PLA230) were emulsified and added at 20% v/v to 50 mM potassium phosphate buffer containing 2% agar. Commercial Impranil DLN-SD emulsions were diluted to 1% (v/v) in the agar. Emulsified agar samples were prepared in 24-well plates. Wells were punched into the agar and enzymes were tested at 50 μg per well (JW45_1534) and 20 μg per well (JW44_1708). JW45_1534 and JW44_1708 reactions were incubated at 50 and 30 °C, respectively, for 4 days to assess emulsion-clearing activity. Impranil clearing plates were incubated at 37 °C for 4 days.

#### PLA10 Powder Clearing Assay

To test activity against solid PLA powder, low-molecular-weight PLA (PLA10, Resomer(R)-202-H) was directly incubated with purified JW45_1534 and JW44_1708 enzymes. Reaction conditions: 15 mg/mL PLA10 powder, 0.25 mg/mL enzyme, and 400 mM ammonium acetate buffer pH 9.0. Reactions and controls lacking an enzyme were setup in triplicate and incubated at 30 and 55 °C for 22 h/1000 rpm for JW44_1708 and JW45_1534, respectively. Following incubation, the reactions were filtered using an Amicon Ultra 0.5 centrifugal filter (3000 MWCO) and centrifuged for 30 min/13,000*g* at room temperature. Lactate oligomers in the filtered samples were analyzed by LC-MS (see the Extended Methods).

#### Buffer Strength Comparison Assay

A comparison of buffer strength was prepared as follows; reactions contained 15 mg/mL PLA10 powder, 0.1 mg/mL enzyme, and 0.5 1, 2, or 3 M ammonium acetate buffer pH 9.0. Reactions and controls lacking enzyme were setup in triplicate and incubated at 30 and 55 °C for 18 h/1000 rpm for JW44_1708 and JW45_1534, respectively. Following incubation, the reactions were filtered using an Amicon Ultra 0.5 centrifugal filter (10,000 MWCO) and centrifuged 30 min/13,000*g* at room temperature. Monomeric lactate in the filtrate was quantified by HPLC. To determine oligomeric lactate production, 5 μL of the filtrate was mixed with 5 μL of 2 M NaOH and incubated at 95 °C for 5 min to hydrolyze lactate oligomers to lactic acid. These NaOH-treated samples were diluted 10-fold and the total lactate concentration was determined by HPLC using external lactate standards. Concentration values were averaged across triplicate samples, errors expressed as standard deviations, and nonenzymatic lactate formation is shown alongside enzymatic lactate formation in the figures.

#### Substrate Loading Assay

To determine the enzyme's activity against high PLA10 powder concentration; reactions contained 9, 15, 36, or 45 mg/mL of untreated PLA10 powder, 0.1 mg/mL enzyme, and 1 M Tris pH 9.0. Reactions and controls lacking enzyme were setup in triplicate and incubated at 30 and 55 °C for 18 h/1000 rpm for JW44_1708 and JW45_1534, respectively. Following incubation, the reactions were processed for HPLC analysis as for the buffer strength comparison assay, with the following differences: To determine oligomeric lactate production, 30 μL of the filtrate was mixed with 30 μL of 2 M NaOH and incubated at 95 °C for 5 min, and the samples were diluted 2.2-fold prior to HPLC analysis.

## Supporting Information Available

Extended methods describing reagent sources, polymer emulsion preparation, high-throughput cloning and expression, additional enzyme assays, and analytical techniques. Supplementary Figures of PCR amplification, high-throughput screening optimization, plate assays for secondary screening, SDS-PAGE gels of purified proteins, substrate crystallinity tests, and alanine mutant testing and comparisons with known enzymes. Supplementary tables containing soil sources, genome sequencing statistics, output from bioinformatic analysis, cloning primers, PCR conditions, Pfam clan tags, and expression optimization conditions (PDF)
