# Supplementary material for: Functional Enrichment and Sequence-Based Discovery Identify Promiscuous and Efficient Poly Lactic Acid Degrading Enzymes
Source: Environ Sci Technol. 2025 Apr 1;59(17):8602–13. doi: 10.1021/acs.est.4c07279 (PMC12060274; doi:10.1021/acs.est.4c07279)
Supplement: Supplementary file 1 — es4c07279_si_001.pdf [file es4c07279_si_001.pdf]

<u>Supporting Information</u>

Functional Enrichment and Sequence-Based Discovery Identify Promiscuous and Efficient Poly Lactic Acid Degrading Enzymes

Gorjan Stojanovski[†,§], Maria Bawn[†,∥], Amy Locks[‡], Esther Ambrose-Dempster[‡], John M. Ward[†], Jack W. E. Jeffries[†,] and Helen C. Hailes[‡,*]*

† = Department of Biochemical Engineering, University College London, Bernard Katz Building, London WC1E 6BT, UK

‡ = Department of Chemistry, University College London, 20 Gordon Street, London WC1H 0AJ, UK

Summary: 50 pages, 11 tables, 12 figures.

Table of Contents

Extended Materials and Methods **3**

1. Reagents **3**

2. Preparation of polyester emulsions **3**

3. Plastic degradation by wild-type strains **3**
3.1 PLA powder strain degradation assay and analysis 3
3.2 PBAT film degradation assay and analysis 4

4. Genomic DNA isolation and whole genome sequencing **4**

5. High-throughput cloning of putative plastic degrading genes **4**
5.1 High-throughput PCR 4
5.2 One-pot restriction ligation cloning and transformation 5
5.3 High-throughput expression of putative plastic degrading enzymes 6

6. Small scale expression and purification for secondary screening of putative PLA degrading enzymes **6**

7. Large-scale protein purification of JW44_1708 and JW45_1534 .... **6**

8. Active site mutants expression and purification **7**

9. Extended Enzyme assays **7**
9.1 Poly-L-lactic acid (PLLA) film assay 7
9.2 Phenylmethylsulfonyl fluoride (PMSF) inhibition assay 8
9.3 Enzyme alanine mutant activity testing 8

10. Analytical techniques **8**
10.1 HPLC analysis of PLA degradation 8
10.2 HPLC analysis of PBAT degradation 9
10.3 LC-MS analysis 9

10.4 Intact protein mass spectrometry 9
10.5 Differential Scanning Calorimetry 10

Supplementary Figures *11*

Figure S1: Overview of the high-throughput cloning workflow. 11

Figure S2: CHAPS lysis reagent creates artifactual zones of clearing. 12

Figure S3: Rare codon usage of putative PDEs. 13

Figure S4: Secondary functional assay of 13 putative PLA degraders. 14

Figure S5: Comparison of amino acid similarity of JW44_1708, JW45_1534 and JW1751_1026 with known PLA and PBAT degrading enzymes. 16

Figure S6: Polyesterase activity screening 17

Figure S7: Visualisation of enzymatic PLA powder degradation assays 18

Figure S8: DSC thermogram of PLA powder and PLLA film. 19

Figure S9: Rotation views of the JW1745_1534 predicted enzyme structure. 20

Figure S10: JW44_1708 active site mutant testing to probe presence of second active site 22

Figure S11: First pass PCR amplification of putative PDEs 23

Figure S12: SDS-PAGE analysis of purified JW44_1708 and JW45_1534 24

Supplementary Tables *25*

Table S1: Source and locations of soil compost samples used in this study. 25

Table S2: Genome sequencing statistics and soil of isolation 26

Table S3: Signal peptide prediction statistics 28

Table S4: Clan annotations of previously characterised PDEs from the PlasticDB. Clans which contain enzymes which degrade PLA/PBAT are highlighted in bold. 29

Table S5: Known PDE Pfam Family-specific functional annotations within clans. 29

Table S6: Table of enzymes tested for functional screening. 30

Table S7: Automated molecular biology pipeline success rates. 36

Table S8: Expression conditions optimisation. 36

Table S9: Table of potential PLA degrading enzymes identified in preliminary functional screen. 37

Table S10: PCR primers. 38

Table S11: PCR amplification conditions for 18 sequences which required optimisation. 47

Enzyme Sequences *48*

References *49*

Extended Materials and Methods

1. Reagents

All chemical reagents were purchased from Merck Millipore or Fisher Scientific and used without further purification. Molecular biology reagents were purchased from NEB. Oligonucleotides were synthesized by Merck Millipore or Fisher Scientific. Poly-(D,L)-lactic acid plastic pellets were obtained from Goodfellow (M_w ~ 55,000 - ME34-GL-000180, M_w ~ 116,000 - ME34-GL-000114, M_w ~ 148,000 - ME34-GL-000124, M_w ~ 230,000 - ME34-GL-000110). Amorphous poly-L-lactic acid (PLLA) film was obtained from Goodfellow (ME34-FM-000100). Low molecular weight poly-(D,L)-lactic acid powder (Resomer®-202 H, acid-terminated, M_w 10,000 – 18,000, 719978) and polycaprolactone (PCL, M_n ~ 80,000, 440744) sodium D,L-lactate and terephthalic acid (TPA) were obtained from Merck Millipore. Ecoflex® polybutylene adipate co-terephthalate (PBAT, 30 µm), polybutylene succinate co-adipate (PBSA, 30 µm), Poly(3-hydroxybutyrate-co-3-hydroxyhexanoate) (PHBH) films were obtained from Biome Bioplastics. Impranil®DLN-SD was obtained from Covestro AG. High molecular weight PBAT pellets (Ecoworld® Biodegradable Polymer) for enrichment cultures were obtained from Jin Hui ZhaoLong High Technology Co. Ltd. Authentic standards of mono(hydroxybutyl)terephthalate (MHBT) and bis(hydroxybutyl)terephthalate (BHBT) were obtained from LGC Standards. Seven soil compost samples were collected between 2019 and 2021 (Table S1).

2. Preparation of polyester emulsions

Polyester plastic (0.25 mg) was dissolved in 20 mL of dichloromethane in a 100 mL Duran bottle. To this was added 0.5 mL of SPAN-80 which was homogeneously mixed using a magnetic stirrer. To this solution was added 50 mL of distilled and deionized H_2O, and the mixture was shaken and sonicated for 10 mins. The dichloromethane was then evaporated by stirring overnight at room temperature. The resulting emulsion was sonicated in a sonication bath for 1 hour to disperse and create a uniform suspension of the polyester plastic.

3. Plastic degradation by wild-type strains

3.1 PLA powder strain degradation assay and analysis.

Degradation of low-molecular weight PLA powder by all strains was determined. PLA powder was dissolved in dichloromethane and then aliquoted into wells of a 96-well plate (giving 10 mg of PLA powder per well) and the solvent was evaporated overnight. Strains were grown overnight in nutrient broth either at 30 °C or 50 °C (depending on their optimal growth temperature) and then diluted to OD_{600} 0.1 in 1.5 mL of nutrient broth in the 96-well plate containing PLA powder. Strains were grown for 48 hours at 37 °C or 50 °C and 50 µL aliquots were taken after 24 hours and 48 hours. Each aliquot was diluted two-fold with 50 µL of dH_2O and filtered using hydrophilic PVDF Durapore membrane filters (47 mm, 0.22 µm pore size, Merck Millipore) in 96-well plate centrifuge filters. The concentration of lactic acid in the filtrate was quantified by HPLC (described in section 10.1), determined using sodium lactate standards. Concentration values were averaged across triplicate samples and errors expressed as standard deviations.

3.2 PBAT film degradation assay and analysis

PBAT degrading strains (JW1739, JW1743, JW1747, JW1748, JW1751, JW1753 and JW1755) were inoculated from a glycerol stock into 3 mL of ¼ strength nutrient broth containing a 1 x 1 cm Ecoflex PBAT film. All cultures were incubated at 37°C static (aside from JW1743 which was incubated at 50 ^{o}C). Controls containing Ecoflex film in ¼ strength nutrient broth were incubated at 37 ^{o}C and 50 ^{o}C for the same timeframe. 200 µL samples were taken after 0, 7, 14 and 21 days of incubation, diluted with an equal volume of 100% DMSO, filtered by centrifuging 30 mins/13000 x *g*/room temperature using Amicon Ultra 0.5 centrifugal filters (MWCO 3000) and the filtrate was analyzed by HPLC (see section 10.2). Reactions were performed in singletons and concentration values of monomer release was quantified using authentic standards of TPA, MHBT and BHBT.

4. Genomic DNA isolation and whole genome sequencing

High molecular weight genomic DNA was isolated by the following method: a single colony of each isolated strain was inoculated in 10 mL of nutrient broth no.2 media and incubated overnight at 37 °C/200 rpm or 50 °C/200 rpm, respectively. The cells were harvested by centrifugation at 9000 x g/10 mins and the pellet was resuspended in 5 mL of TE buffer (10 mM Tris-HCl pH 7.5, 1 mM EDTA). Lysozyme (final concentration: 50 µg/mL) was added and the cells were incubated at 37 °C without shaking for 30 mins. Sodium dodecyl sulfate (final concentration: 1%) and pronase (final concentration: 50 µg/mL) were added and the mixture was incubated at 37 °C for 2 hours. RNAse (final concentration: 20 µg/mL) was added and the solution incubated at 50 °C for 1 hour. 0.5 mL of 5 M NaCl solution was added followed by 10 mL of 100 % EtOH. The DNA precipitate was spooled onto a sterile inoculation loop, transferred to 5 mL of 10 mM Tris-HCl pH 8.5 and left to dissolve. DNA preparation quality was assessed by Nanodrop quantification and restriction enzyme digestion followed by agarose gel electrophoresis. Whole-genome sequencing and contig assembly was performed by MicrobesNG on an Illumina HiSeq platform. The closest available reference genome was identified using *Kraken*, and the reads were mapped to this using *BWAmem* to assess the quality of the data. De novo assembly of the reads was performed using *SPAdes*, and the reads mapped onto the resultant contigs, again using *BWAmem* to produce quality metrics. Automated gene annotation was performed using Prokka. Genome sequencing statistics are summarized in Table S2.

5. High-throughput cloning of putative plastic degrading genes

5.1 High-throughput PCR

Primers for amplification of the 105 putative enzymes from genomic DNA were designed *in silico* to remove the signal sequence predicted by SignalP 6.0[1] and replace it with an ATG codon. Primers were designed using a custom python script to ensure similar melting temperatures and synthesized by Merck Millipore (Table S10). PCR amplification was performed in a 96-well plate using an openTrons OT-2 liquid handling robot, fitted with GEN2 P300 and GEN2 P20 8-channel pipette heads. The PCR reaction conditions were as follows: 1 µL of genomic DNA, 4 µL 10 µM forward + reverse primers (final concentration – 1 µM of each primer), 4 µL 100% DMSO (final concentration - 10%), 11 µL nuclease-free H_2O, 20 µL 2x Phusion PCR master mix + High Fidelity buffer. For GC-rich amplicons defined as sequences with a GC content

> 60%, 20 µL of 2x Phusion PCR master mix + GC buffer was used instead. The plate was sealed with a Microseal B adhesive seal (Bio-Rad) and thermocycling conditions were as follows: Initial denaturation – 98 °C for 5 mins, 30 cycles of Denaturation – 98 °C for 10 secs, Annealing – 61 °C for 30 secs, Extension – 72 °C for 90 secs, Final extension – 72 °C for 7 min. Under these conditions, 74 genes were amplified as single bands and purified using the Charge-Switch PCR Clean-up kit (ThermoFisher Scientific), 13 bands were purified by gel extraction using the Monarch Gel Extraction kit (NEB) and 12 bands did not amplify (Figure S11).

For the 12 genes of interest which did not amplify and the 6 genes from strain JW1740, The PCR conditions were modified to obtain successful PCR amplification (Table S11). Two gene sequences were not successfully amplified and were excluded from downstream analysis.

5.2 One-pot restriction ligation cloning and transformation

Purified gene amplicons were digested and ligated into pET29a(+) using plasmids pET29a(+)-sacB-*SapI* and pET29a(+)-sacB-*BsaI* in one-pot as previously reported[2] in high-throughput using an openTrons OT-2 liquid handling robot. The restriction-ligation conditions were as follows – 1 µL of insert DNA, 1 µL of pET29:sacB-*SapI* vector (100 ng), 1 µL T4 DNA ligase, 0.5 µL *SapI*, 1 µL T4 DNA ligase buffer, 6.5 µL nuclease-free H_2O. All sequences containing *SapI* cut sites were cloned using pET29:sacB-*BsaI* and 1 µL of *BsaI*. Restriction-ligation reactions were incubated at 37 °C for 1 hour and then overnight at 4 °C.

Enzymes (4 genes) which contained both *SapI* and *BsaI* cut sites were cloned using circular polymerase extension cloning (CPEC). pET29a(+) was linearized and the genes were amplified by PCR (Table S10) and then used for CPEC, reactions conditions – 100 ng of linearized pET29a(+) vector, 100 – 150 ng of insert DNA, 10% DMSO, 1 x Phusion PCR master mix + High Fidelity buffer. CPEC thermocycling conditions were as follows: Initial denaturation – 98 °C for 1 min, 18 cycles of Denaturation – 98 °C for 30 secs, Annealing – 52 °C for 30 secs, Extension – 72 °C for 150 secs, Final extension – 72 °C for 7 min.

Restriction-ligation reactions and CPEC reactions were transformed into *E. coli* NovaBlue in high-throughput using an openTrons OT-2 liquid handling robot. 2 µL of ligation reactions or 5 µL of CPEC reactions were mixed with 20 µL of competent cells in a 96-well PCR plate and incubated at 4 °C on an OT-2 GEN2 Temperature module for 30 min with a PCR plate attachment. The samples were heat-shocked at 42 °C in a water bath for 45 sec and then returned to 4 °C on the temperature module for 5 min. 150 µL of SOC media (Invitrogen) was added and the cells were recovered at 37 °C/1000 rpm for 1 hour. The plate was centrifuged 2000 x *g*/1 min and 125 µL of SOC media was removed. The cells were resuspended in the remaining media and plated onto selective agar media in 24 well plates. Plates were spread using sterile glass beads and incubated overnight at 37 °C. The selective media for genes cloned into pET29:sacB-*SapI* or pET29:sacB-*BsaI* was LB + 10% sucrose + 50 mg/mL kanamycin. For genes cloned by CPEC, the selective media was LB + 50 mg/mL kanamycin.

Successfully cloned genes were confirmed by plasmid isolation using a Qiaprep Spin mini-prep kit (QIAGEN) and Sanger DNA sequencing performed by Eurofins Genomics. Six enzymes were not successfully cloned, giving a final set of 97 enzymes for functional screening.

5.3 High-throughput expression of putative plastic degrading enzymes

Cloned plasmids were transformed into *E. coli* Rosetta-gami-2 (DE3) cells using an openTrons robot and directly inoculated into 250 µL of LB media containing 50 µg/mL kanamycin and 20 µg/mL of chloramphenicol in a 2.2 mL 96 deep-well plate (Abgene AB-0932), sealed with a Breathe Easier plate seal (BERM-2000, Diversified Biotech) and incubated for 17 hours at 37 °C/1800 rpm on a thermomixer. Enzymes were expressed in MagicMedia auto-induction media (ThermoFisher Scientific) and in Terrific Broth (Merck Millipore). Overnight cultures were inoculated to an OD ~ 0.1 in 250 µL of MagicMedia or 500 µL of Terrific broth in a 2.2 mL 96-well deep-well plates, containing 50 µg/mL kanamycin and 20 mg/mL chloramphenicol. Expression plates were sealed with a Breathe Easier plate seal and incubated at 37 °C/1500 rpm for 5 hours (for TB cultures) or 37 °C/1800 rpm for 3 hours (for MagicMedia cultures) on a thermomixer. After 3 hours of incubation, MagicMedia cultures were transferred to 25 °C/1800 rpm and expressed for 21 hours. After 5 hours, the TB cultures were induced with 0.5 mM IPTG and incubated at 25 °C/1500 rpm for 21 hours.

Cultures were harvested by centrifuging 4400 x *g*/4 °C for 20 mins and cell pellets were resuspended in lysis buffer (1 x BugBuster (Merck Millipore), 50 mM potassium phosphate buffer pH 8.0, 25 U/mL Benzonase (Merck Millipore), 150 mM NaCl) and incubated for 30 mins/1200 rpm at room temperature on a thermomixer. The cell lysates were clarified by centrifuging 4400 x *g*/4 °C /30 mins.

6. Small scale expression and purification for secondary screening of putative PLA degrading enzymes

For secondary confirmation of putative PDEs, single colonies of *E. coli* Rosetta-gami-2(DE3) cells containing potential hits from primary screening were inoculated into 10 mL of LB and incubated at 37 °C/250 rpm for 18 hours. Starter cultures were then inoculated to an OD600 ~ 0.1 in 50 mL of MagicMedia containing 50 µg/mL kanamycin and 20 µg/mL of chloramphenicol and incubated at 37 °C/250 rpm for 3 hours, then expressed at 25 °C/265 rpm for 24 - 48 hours. Cultures were harvested by centrifuging 10000 x *g*/10 mins/4 °C and lysed in lysis buffer (50 mM sodium phosphate pH 8.0, 300 mM sodium chloride, 10 mM imidazole, 1 x BugBuster, 25 U/mL Benzonase) by incubation on a rocking shaker for 30 mins and then clarified by centrifuging 20000 x g/30 mins/4 °C. Proteins were then purified small scale using Pierce High-Capacity Ni-NTA magnetic beads (ThermoFisher) at 4 °C following the manufacturer's instructions. Protein concentration and purity were assessed by a Bradford assay and SDS-PAGE, respectively. The crudely purified proteins were tested for PLA emulsion clearing activity in an agar-plate clearing assay. The agar test plate contained 2% agar + 50 mM potassium phosphate buffer pH 8.0 and 10% (v/v) emulsified PLA. Wells were punched into the agar plate using a sterile hole borer and 30 µL of protein sample was loaded into the wells. Plates were incubated at 37 °C for 48 hours to assess enzymatic emulsion clearing activity.

7. Large-scale protein purification of JW44_1708 and JW45_1534

For upscaled protein expression, single colonies of *E. coli* Rosetta-gami-2 (DE3) cells containing enzymes JW45_1534 or JW44_1708 were inoculated into 10 mL of LB and incubated at 37 °C /250rpm for 18 hours. Starter cultures were then inoculated to an OD600 ~ 0.1 in 500 mL of MagicMedia containing 50 µg/mL kanamycin and 20 µg/mL of chloramphenicol and incubated at 37 °C/250 rpm for 3.5 hours, then expressed at

25 °C /275 rpm for 24 hours. Cultures were harvested by centrifuging 10000 x *g*/10 mins/4 °C in a Beckman Coulter Ultracentrifuge fitted with JA-10 rotor. Cell pellets were lysed in lysis buffer (50 mM sodium phosphate pH 8.0, 300 mM sodium chloride, 10 mM imidazole, 1 x BugBuster, 25 U/mL Benzonase) by incubation on a rocking shaker for 30 mins and then clarified by centrifuging 20133 x *g*/30 mins/4 °C. Proteins were then purified via the C-terminal 6x-Histag by immobilized metal ion affinity chromatography using a vacuum-manifold and 3 mL Ni^{2+}-charged chelating Sepharose Fast Flow (GE Healthcare) resins, pre-equilibrated with purification buffer (50 mM sodium phosphate pH 8.0, 300 mM sodium chloride, 10 mM imidazole). Proteins were washed with purification buffer containing a stepwise gradient of imidazole [20 mM (30 mL), 50 mM (30 mL), 100 mM (18 mL)] prior to elution with purification buffer containing 500 mM imidazole (10 mL). Protein elution was monitored by a Bradford assay and protein purity was assessed by SDS-PAGE (Figure S12). Following elution, proteins were buffer-exchanged into 50 mM sodium phosphate buffer, 100 mM NaCl using Amicon Ultracel centrifugal concentrators, glycerol was added to a final concentration of 10%, protein solutions were snap-frozen in liquid N_2 and stored at –80 °C.

8. Active site mutants expression and purification

Alphafold2 structures of JW45_1534 and JW44_1708 were predicted using ColabFold[3]. After analysis of the predicted structures, the following alanine mutants of JW44_1708 were synthesized – JW44_1708 S77A, JW44_1708 S120A and subcloned into pET29a(+) by Genscript. The plasmids were transformed into *E. coli* Rosetta-gami 2 (DE3) and expressed as follows; Single colonies were inoculated into 10 mL of LB and incubated at 37 °C/250 rpm for 18 hours. Starter cultures were then inoculated to an OD600 ~ 0.1 in 50 mL of MagicMedia containing 50 µg/mL kanamycin and 20 µg/mL of chloramphenicol and incubated at 37 °C/250 rpm for 3 hours, then expressed at 25 °C/265 rpm for 24 - 48 hours. Cultures were harvested by centrifuging 10000 x *g*/10 mins/4 °C and lysed in lysis buffer (50 mM sodium phosphate pH 8.0, 300 mM sodium chloride, 10 mM imidazole, 1 x BugBuster, 25 U/mL Benzonase) by incubation on a rocking shaker for 30 mins and then clarified by centrifuging 20000 x *g*/30 mins/4 °C. Proteins were then purified via the C-terminal 6x-Histag by IMAC as described above, but eluting into 50 mM Tris-HCl pH 8.0, 300 mM sodium chloride, 500 mM imidazole. Proteins were buffer-exchanged into 50 mM Tris-HCl pH 8.0, 100 mM NaCl using Amicon Ultracel (3000 MWCO) centrifugal concentrators, glycerol was added to a final concentration of 10%, protein solutions were snap-frozen in liquid N_2 and stored at –80 °C.

9. Extended Enzyme assays

9.1 Poly-L-lactic acid (PLLA) film assay

To determine activity against PLLA film; reactions contained ~ 3.6 mg of PLLA film, 0.1 mg/mL enzyme and 1 M ammonium acetate buffer pH 9.0, in a final reaction volume of 250 uL. Reactions and controls lacking enzyme were set-up in triplicate and incubated at 30 °C and 55 °C for 18 hours/1000 rpm for JW44_1708 and JW45_1534, respectively. Following incubation, the reactions were filtered using a Vivaspin 500 centrifugal filter (10000 MWCO) and centrifuged 20 mins/12000 x g at 4 °C. Monomeric lactate in the filtrate was quantified by HPLC. To determine oligomeric lactate production, 30 µL of the filtrate was mixed with 30 µL of 2 M NaOH and

incubated at 95 °C for 5 mins to hydrolyze lactate oligomers to lactic acid. These NaOH treated samples were diluted 2.2-fold and total lactate concentration was determined by HPLC using external lactate standards.

9.2 Phenylmethylsulfonyl fluoride (PMSF) inhibition assay

JW44_1708 inhibition at various pH values was determined using a turbidimetric emulsion clearing assay. Prior to reaction, PMSF (100 mM prepared in 100% IPA) was added to a solution of JW44_1708 (final PMSF concentration of 1 mM) and incubated at room temperature for 15 mins in sodium phosphate buffer pH 8.0 to covalently inhibit the catalytic serine. To account for solvent effects, an equivalent volume of IPA was added to the uninhibited JW44_1708 reaction and it was incubated for the same time prior to activity assessment. Reaction conditions were as follows; 100 mM buffer, 20% (v/v) low-molecular weight Resomer(R) 202-H PLA emulsion (PLA10), 0.1 mg/mL enzyme. The following buffers were used to cover different pH ranges: MES pH 6.5, Tris pH 7.0 - 9.0, Glycine-NaOH pH 9.5. The reactions and control lacking enzyme were set-up in triplicate, in a 96-well plate. Reactions were incubated for 2 hours at 37 °C/300 rpm in a plate reader (CLARIOstar Plus Microplate Reader, BMG LabTech), monitoring absorbance at 580 nm every 80 secs. Absorbance values after 30 mins of incubation were averaged across replicates and compared relative to the no enzyme control to quantify extent of plastic emulsion clearing. PMSF labelled and unlabeled proteins were also analyzed by intact protein mass spectrometry.

9.3 Enzyme alanine mutant activity testing

Activity of the active site mutants was assessed at pH 6.5 - 9.5 using a turbidimetric emulsion clearing assay. Reaction conditions were as follows; 100 mM buffer, 20% (v/v) low-molecular weight Resomer(R) 202-H PLA emulsion (PLA10), 0.05 mg/mL enzyme. The following buffers were used to cover different pH ranges: MES pH 6.5, Tris pH 7.0 - 9.0, Glycine-NaOH pH 9.5. The reactions and control lacking enzyme were set-up in triplicate, in a 96-well plate. Reactions were incubated for 2 hours at 37 °C/300 rpm in a plate reader (CLARIOstar Plus Microplate Reader, BMG LabTech), monitoring absorbance at 580 nm every 90 secs. Absorbance values after 30 mins of incubation were averaged across replicates and compared relative to the no enzyme control to quantify extent of plastic emulsion clearing.

10. Analytical techniques

10.1 HPLC analysis of PLA degradation

HPLC analysis was performed using a Dionex Ultimate 3000 HPLC System, with a Dionex Ultimate 3000 RS Pump, a Dionex Ultimate 3000 Autosampler, a Dionex Ultimate 3000 Column Compartment and an Ultimate 3000 RS Diode Array Detector and (ThermoFisher Scientific, US). Separations were performed on an Aminex HPX87-H column (300 mm × 7.8 mm, 9 µm particle size, Bio-Rad). Monomeric products were quantified using a 25-minute isocratic method with a mobile phase of 5 mM H_2SO_4 (degassed with Helium) at a flow rate of 0.55 mL/min. The column temperature was set to 60 °C, sample tray temperature at 4 °C, injection volume was 10 µL and absorbance was measured at 214 nm. Under these conditions, lactic acid eluted at 14 – 15 mins, and lactamide at 19 – 20 mins. Lactic acid was quantified using

a linear calibration curve from 0.156 - 20 mg/mL, and lactamide was quantified using standards at 0.156 - 10 mg/mL.

10.2 HPLC analysis of PBAT degradation

HPLC set-up was the same as in section 10.1. Separations were performed on an ACE-C18 RP column (150 mm × 4.6 mm, 5 µm particle size, ACE). Monomeric products were quantified using a 10-minute gradient method with mobile phase A – 0.1% formic acid, mobile phase B – acetonitrile at a flow rate of 1 mL/min. Method: 16% B for 2 mins, 16 – 50% B gradient for 1 min, 50% B hold for 1 min, 50 - 95% B for 1 min, 16% B for 5 mins to re-equilibrate. The column temperature was set to 30°C, injection volume was 10 µL and absorbance was measured at 245 nm. Under these conditions, TPA eluted at 3.1 mins, and MHBT at 5.4 mins and BHBT at 5.7 mins. All standards were quantified using linear calibration curves from 6.25 – 100 µM.

10.3 LC-MS analysis

Filtered reactions were analyzed without prior dilution using a ThermoScientific Vanquish ISQ EC single quadrupole mass spectrometer fitted with Vanquish Binary Pump F, a Vanquish Split Sampler FT, a Vanquish Variable Wavelength Detector F, and a Hypersil GOLD C18 column (150 mm x 2.1 mm, 1.9 µm particle size, ThermoScientific). Samples were analyzed using a 22 minute gradient method with mobile phase A of 0.1% formic acid in MilliQ H2O and mobile phase B of acetonitrile - 0 - 4 mins (2% B), 4 - 15 mins (2 - 70% B), 15 – 18 mins (70% B), 18 – 18.1 mins (70% B – 2% B), 18.1 – 22 mins (2% B). MS detection was performed in negative ESI full scan mode, with a m/z range of 10 – 1000 and a source CID voltage of 20. Extracted ion chromatograms were produced from this full scan for the masses of PLA oligomers as previously reported[4] - *m/z* 89.1 (lactic acid), 161.1 (lactate dimer), 233.1 (lactate trimer), 305.1 (lactate tetramer), 377.1 (lactate pentamer), 449.1 (lactate hexamer), 521.2 (lactate heptamer), 593.2 (lactate octamer), 665.2 (lactate nonamer).

10.4 Intact protein mass spectrometry

To analyze extent of PMSF labelling by mass spectrometry, the protein samples were prepared as follows. JW45_1534 and JW44_1708 proteins were diluted to 0.5 mg/mL and split into equal fractions. To one fraction of each protein, 1 µL of 100 mM PMSF was added and all samples were incubated at room temperature for 15 mins. Zebaspin desalting columns (0.5 mL, 7k MWCO, ThermoScientific) were centrifuged 1500 x *g*/4 °C/1 minute to remove the storage solution and equilibrated 3 x with 300 µL of 20 mM ammonium formate buffer pH 8.0. The protein solutions were then loaded onto the Zebaspin desalting columns, centrifuged 1500 x g/4 °C/1 minute and collected in a new tube. The eluted solution was then re-loaded onto the column twice and then analyzed by LC-MS.

The samples were analyzed on the Agilent 6510 QTOF LC-MS system in the UCL Chemistry Mass Spectrometry Facility. Ten µL of each sample (around 0.2 mg/mL protein concentration) was injected onto a PLRP-S column (150 mm x 2.1 mm, 8 µm particle size, 1000Å, Agilent), which was maintained at 60 °C. The separation was achieved using mobile phase A (water with 0.1% formic acid) and B (acetonitrile, with 0.1% formic acid) using a gradient elution at a flow rate of 0.3 mL/min. The column effluent was continuously electrosprayed into the capillary ESI source of the Agilent

6510 QTOF mass spectrometer. ESI mass spectra were acquired in positive electrospray ionization (ESI) mode at the m/z range 1,000 - 3,200 in profile mode. The raw data was converted to zero charge mass spectra using maximum entropy deconvolution algorithm within the MassHunter software version B.07.00.

10.5 Differential Scanning Calorimetry

DSC analysis was used to assess crystallinity of the PLLA film and PLA powder. DSC analysis was performed using a Multi-Sample X3 DSC (TA Instruments, USA), calibrated with indium (T_m = 156.6 °C, ΔH_f = 28.71 J/g). The analysis was performed in a nitrogen atmosphere with a base purge rate of 300 mL/min and a cell purge rate of 50 mL/min. Approx. 5 mg of the PLA sample was loaded into a T_{zero} aluminium pan and crimped with a T_{zero} lid with pinhole. A standard heating ramp at 10 ºC/min from -70 ºC to 250 ºC was employed. Trios software (TA Instruments) was used for analysis.

Supplementary Figures

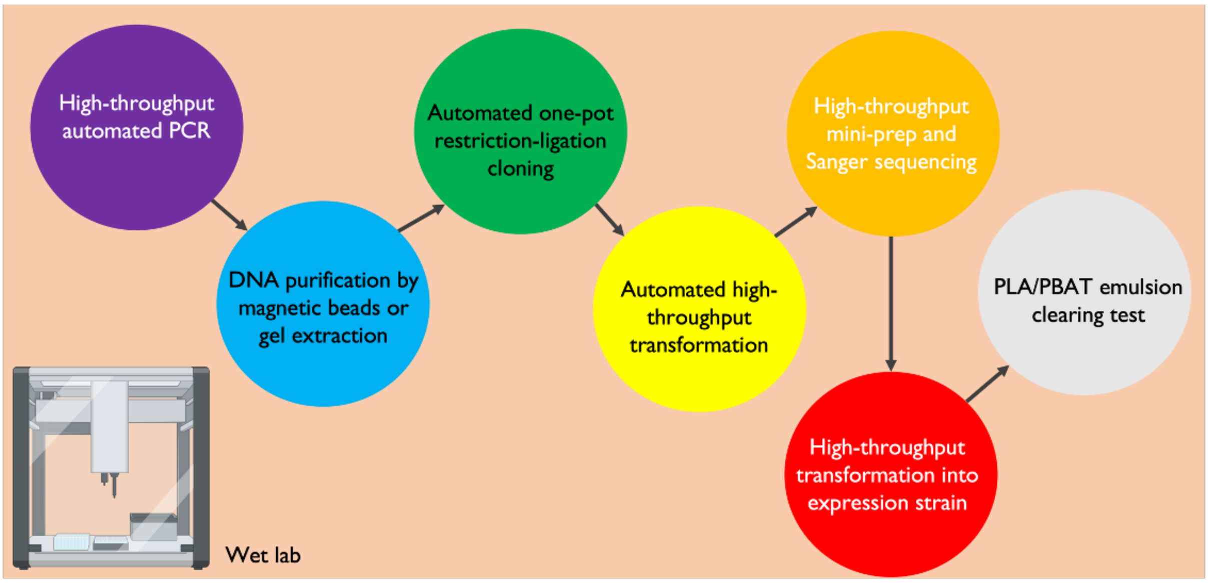

Figure S1: Overview of the high-throughput cloning workflow.

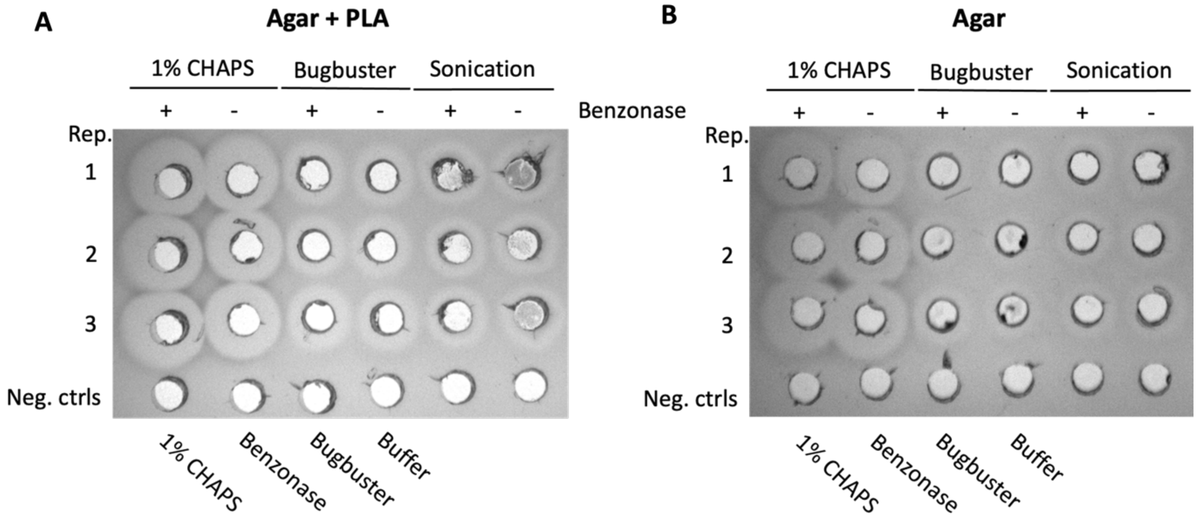

Figure S2: CHAPS lysis reagent creates artifactual zones of clearing. (A) 0.1% w/v Emulsified PLA plate. *E. coli* T7 Shuffle cells expressing pET29a(+) empty vector were lysed using 1% CHAPS, 1x BugBuster or by sonication, and the clarified lysate was loaded into each of the wells. For each lysis conditions, Benzonase was either omitted or added at 2.5U/mL. As negative controls, Solutions of each of the lysis reagents in phosphate buffer pH 8.0 was added to the wells. When cells are lysed with CHAPS, large zones are observed, which are smaller with other lysis conditions. Lysing with BugBuster removed the presence of these artifactual zones during functional screening. (B) Same as for (A) only on agar plates containing no emulsified polymer.

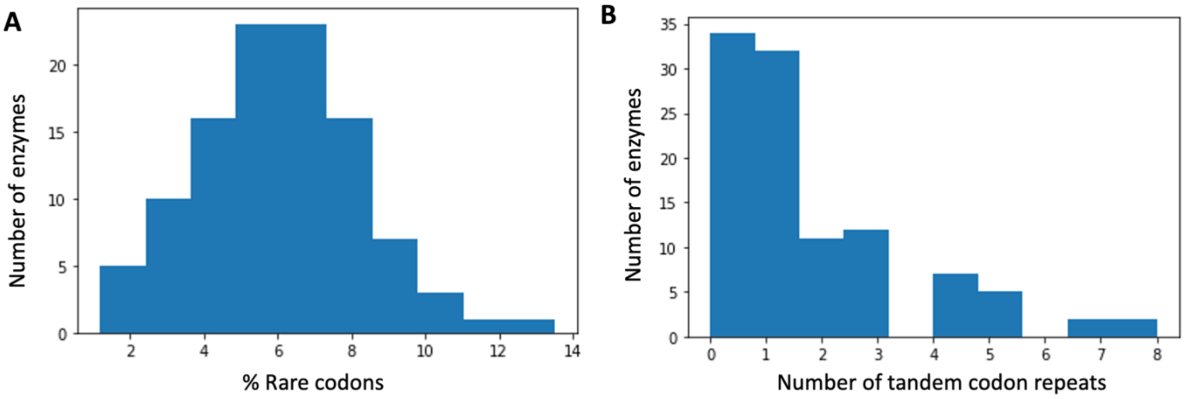

Figure S3: Rare codon usage of putative PDEs. (A) Percentage of rare codons in the 105 PDEs functionally screened. Rare codons were defined as the seven rare codons within *E. coli* (CTA, CCC, CGA, ATA, AGA, AGG, GGA) and percentages are defined as the frequency of rare codons as a percentage of total gene length. (B) Occurrence of tandem rare codon repeats. Tandem rare codon repeats are defined as the number of consecutive double rare codon occurrences in each enzyme sequence.

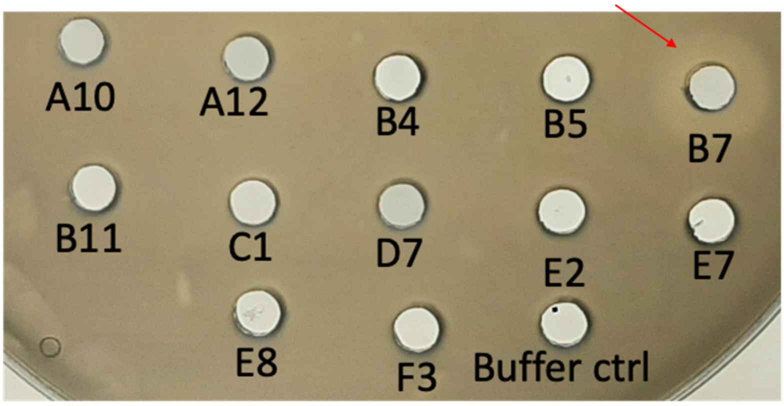

Figure S4: Secondary functional assay of 13 putative PLA degraders. Each of enzymes in each well (see Table S9 for enzyme details) was expressed small scale and purified using magnetic beads and re-tested for zone clearing activity against emulsified PLA (0.1% w/v). Image is representative of duplicate experiments.

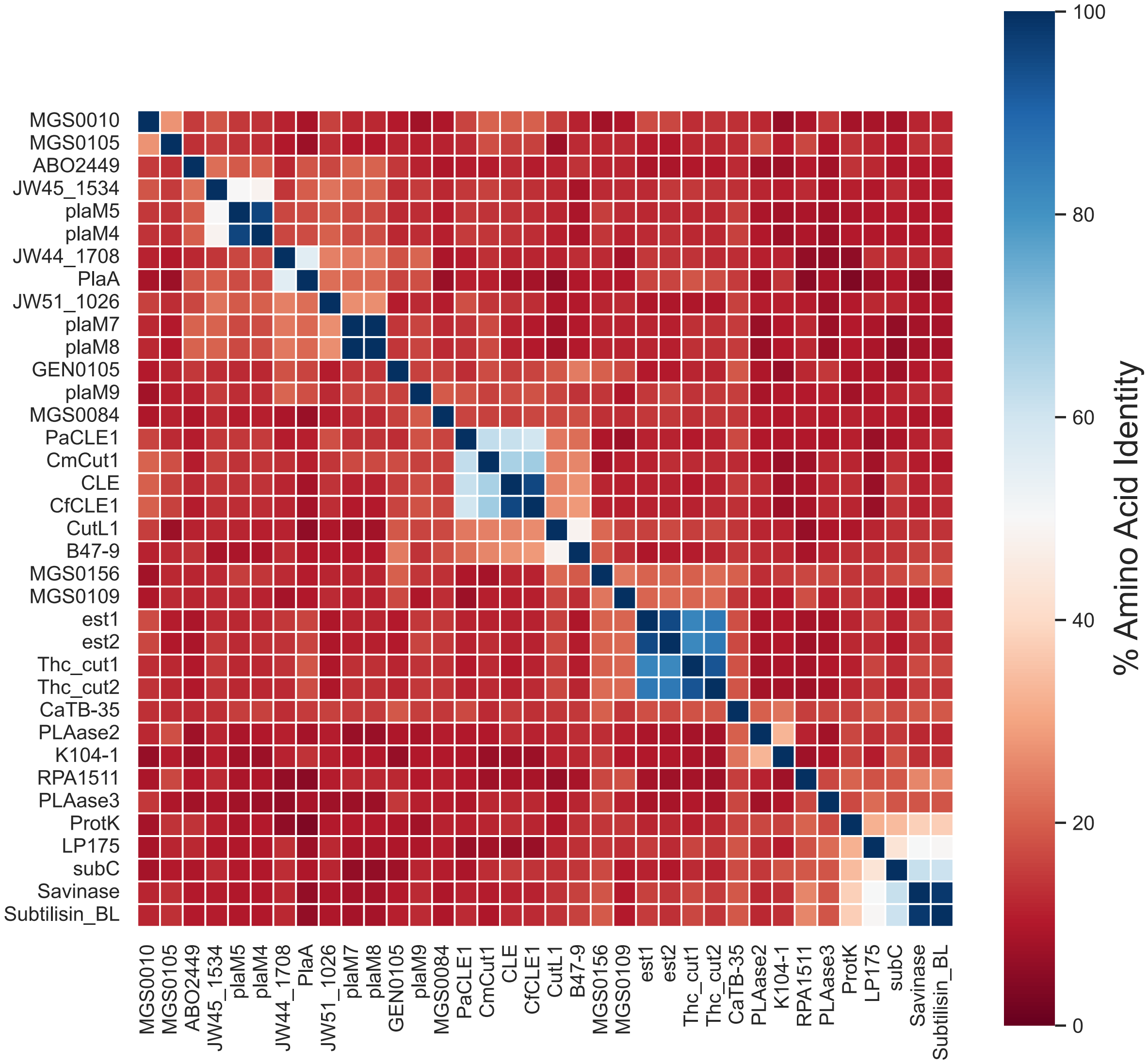

Figure S5 continued on next page.

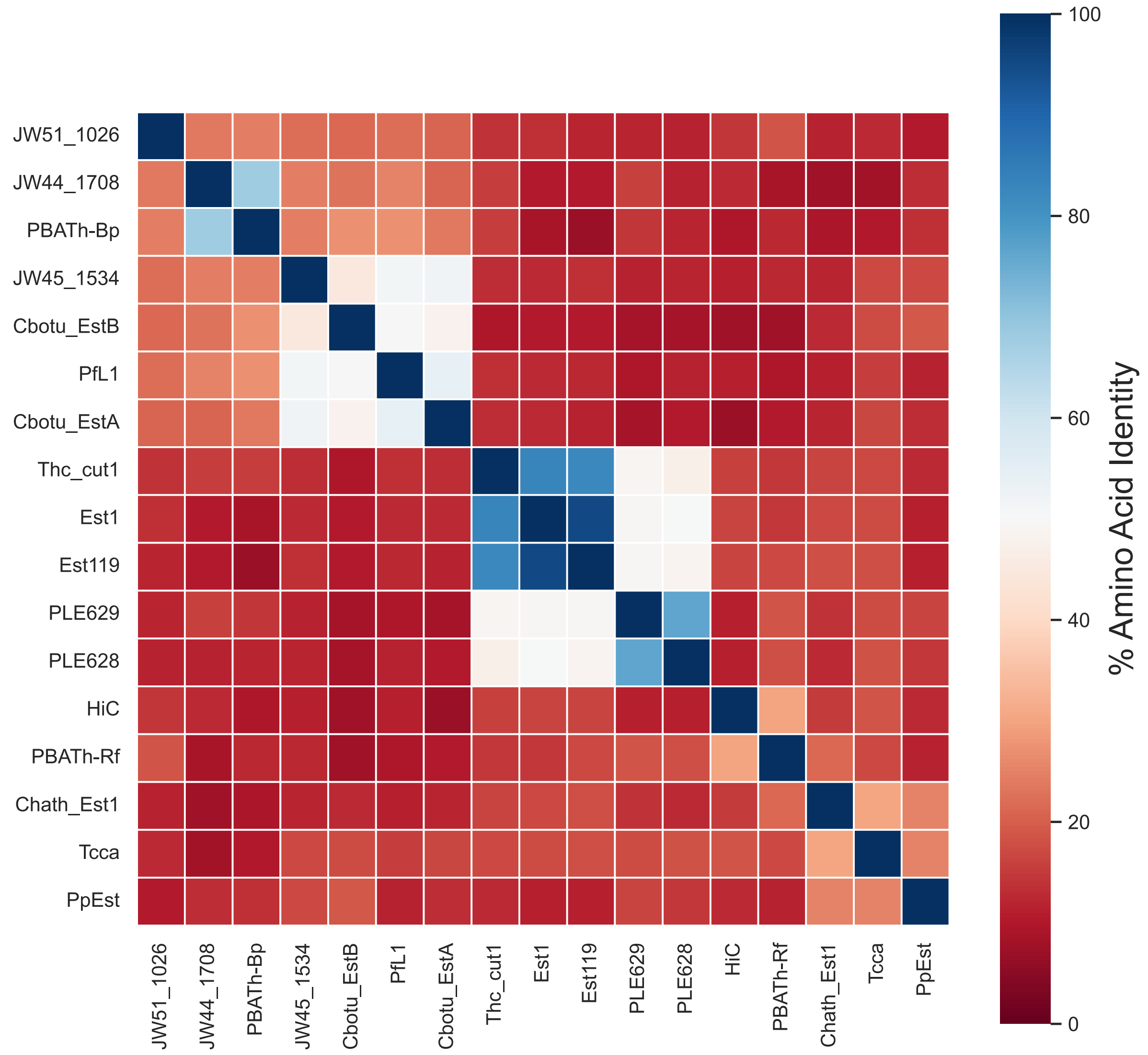

Figure S5: Comparison of amino acid similarity of JW44_1708, JW45_1534 and JW1751_1026 with known PLA and PBAT degrading enzymes. The first heatmap compares the enzymes identified in this study with known PLA degrading enzymes, the second heatmap compares the enzymes with known PBAT degrading enzymes. All enzyme sequences were obtained from the Plastics-Active Enzymes Database (PAZY) Sequences were obtained from the sources cited in PAZY[4–23]. Signal peptides and cloning/purification tags were removed from the sequences prior to amino acid identity determination.

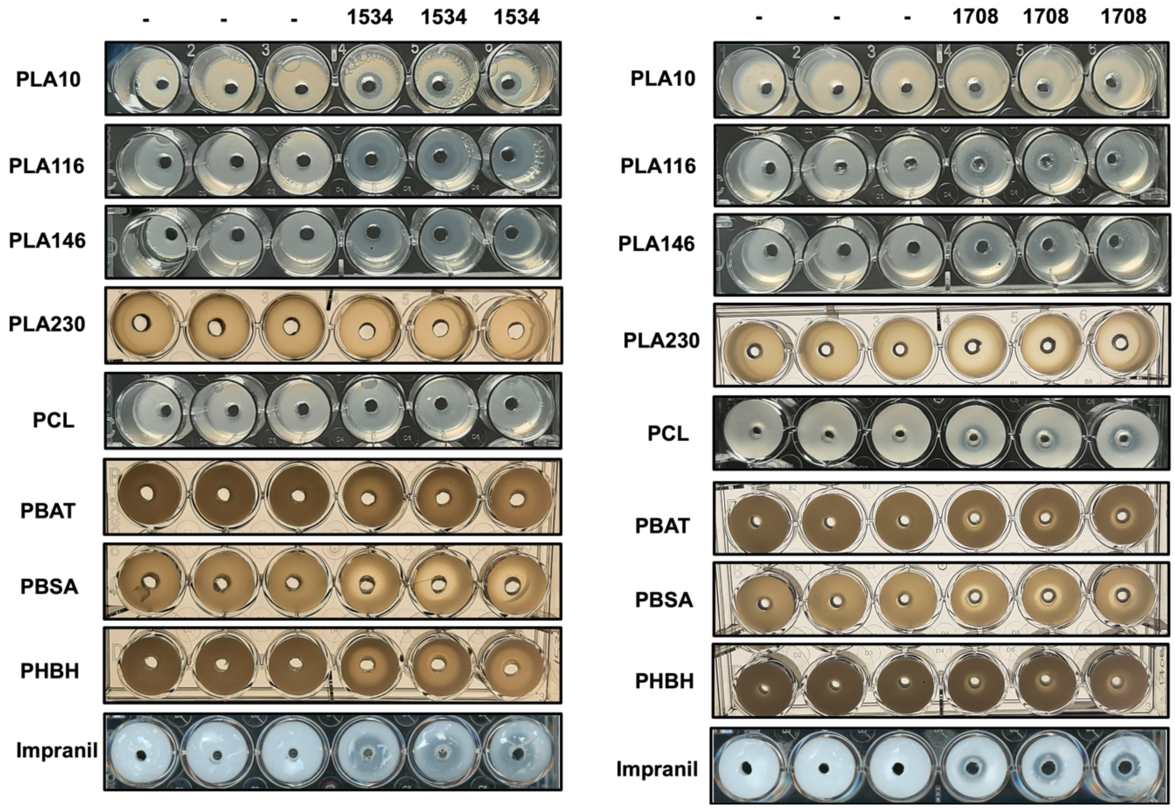

Figure S6: Polyesterase activity screening. 20 µg/well of purified JW44_1708 and 50 µg/well of purified JW45_1534 in 50 mM phosphate buffer pH 8.0 was tested in triplicate against each polymer displayed. Buffer only controls (-) were also performed in triplicate for each enzyme. Emulsified agar plates contained 50 mM potassium phosphate buffer pH 8.0 as a pH buffering agent. Plates were incubated at 50 °C for JW45_1534 and 30°C for JW44_1708, respectively for 48 hours prior to imaging. Impranil reactions were incubated at 37 °C for 48 hours. PLA10 = polylactide (M_w ~ 10,000), PLA55 = polylactide (M_w ~ 55,000), PLA116 = polylactide (M_w ~ 116,000), PLA148 = polylactide (M_w ~ 148,000), PLA230 = polylactide (M_w ~ 230,000), PBAT = polybutylene terephthalate co-adipate, PCL = polycaprolactone, PHBH = Poly(3-hydroxybutyrate-co-3-hydroxyhexanoate), Impranil = Impranil DLN SD.

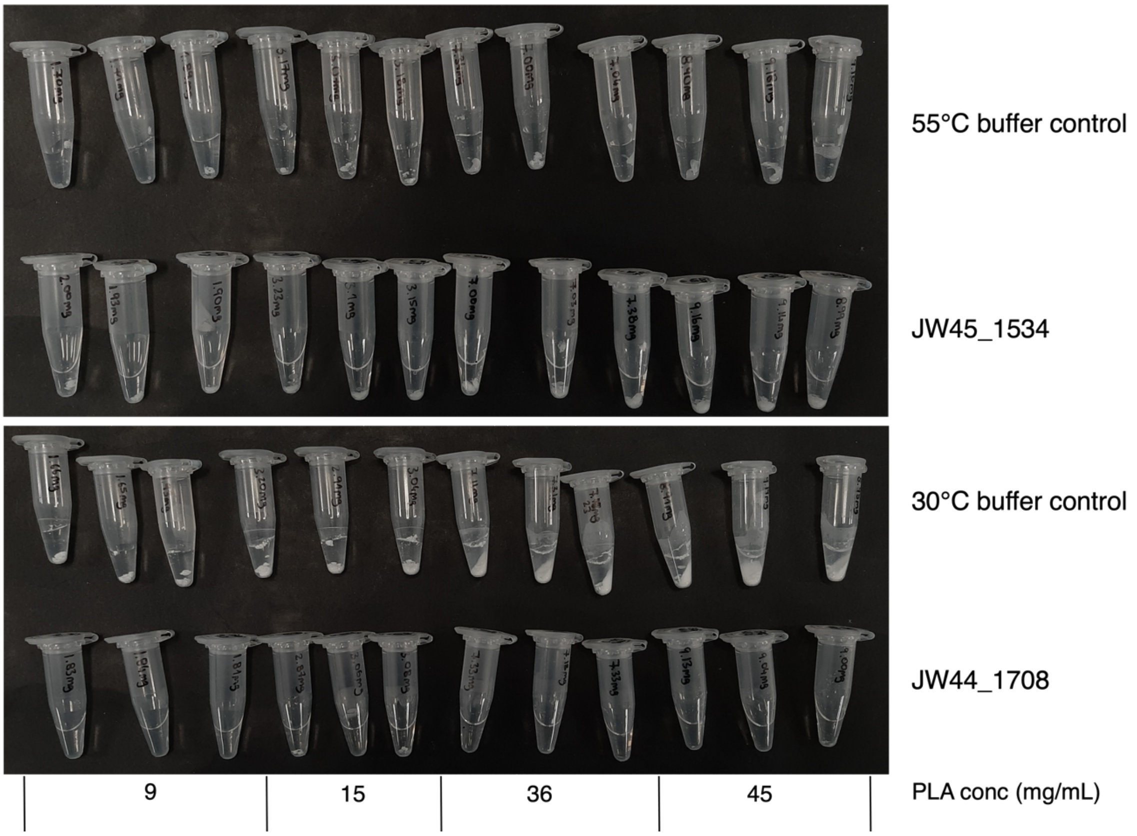

Figure S7: Visualisation of enzymatic PLA powder degradation assays. Images were taken 22 hours post reaction. In contrast to the no enzyme control at 30 °C, no solid residues were observed at all PLA concentrations aside from small amounts at 15 mg/mL PLA for JW44_1708. However solid powder was still present after reaction with JW45_1534 at all tested concentrations, comparable to the no enzyme control.

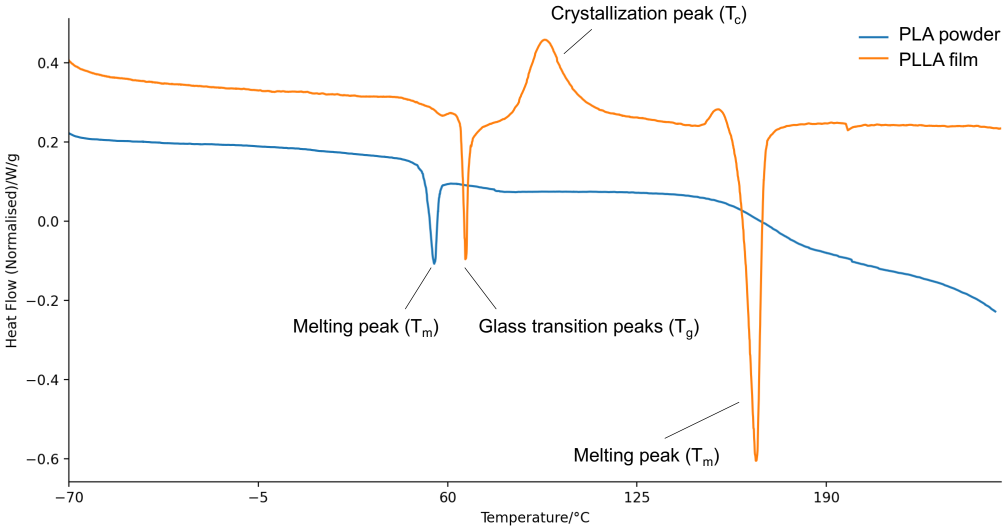

Figure S8: DSC thermogram of PLA powder and PLLA film.

Heating rate 10 ºC/min; temperature range -70 to 250 ºC; under nitrogen atmosphere. PLLA film: T_g = 66 ºC, T_c = 93 ºC, T_m = 166 ºC, PLA powder: T_g = 55 ºC. At first heat, the PLLA film sample used in the study had a glass transition temperature of 66.0 °C, a cold crystallization temperature of 93.2 °C, a T_m of 166.1 °C, a ΔH_{cc} of 18.9 J g^{-1}, a ΔH_m of 28.12 J g^{-1} and an X_c of 10%, calculated using a reference melting peak value of 93.7 J g^{-1} for 100% crystalline PLA[24]. The peak at 58 °C is the melting peak for the PLA powder used in this study.

A

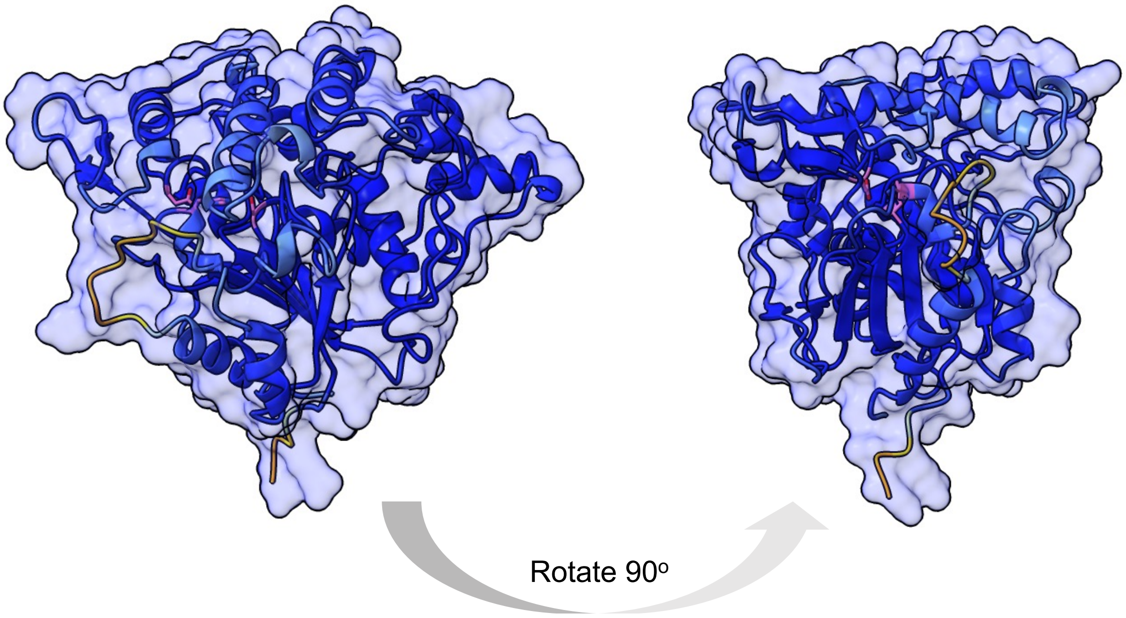

Figure S9: Rotation views of the JW1745_1534 predicted enzyme structure.
The residues coloured in pink are the core catalytic triad. The two views further demonstrate that the active site is buried within the core of JW145_1534. Figure was prepared using Chimera X.

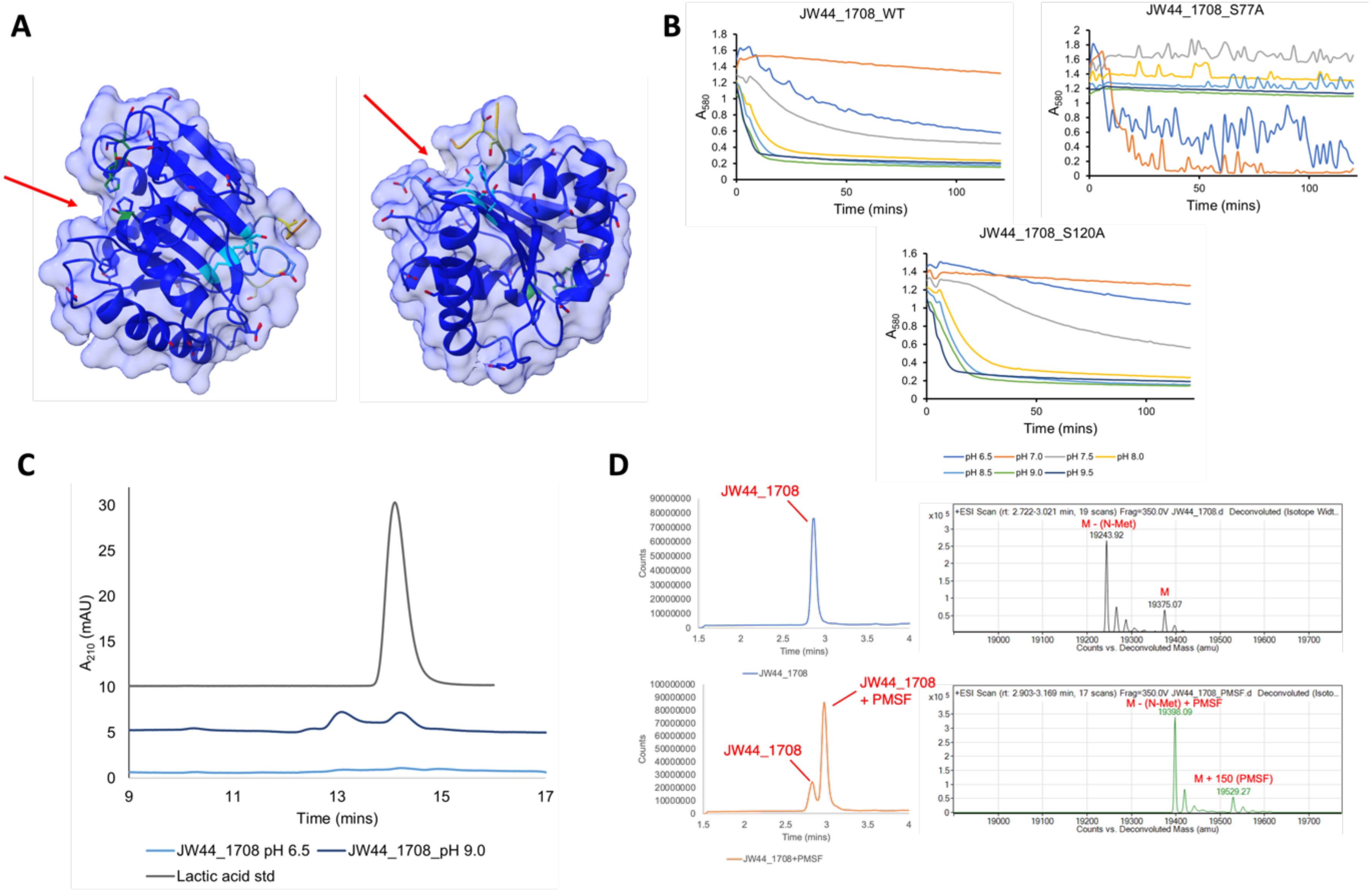
A
B
JW44_1708_WT
JW44_1708_S77A
JW44_1708_S120A
A580
Time (mins)
pH 6.5
pH 7.0
pH 7.5
pH 8.0
pH 8.5
pH 9.0
pH 9.5
C
A210 (mAU)
Time (mins)
JW44_1708 pH 6.5
JW44_1708_pH 9.0
Lactic acid std
D
JW44_1708
JW44_1708 + PMSF
Counts
Time (mins)
JW44_1708+PMSF
M - (N-Met)
19243.92
M
19375.07
M - (N-Met) + PMSF
19398.09
M + 150 (PMSF)
19529.27
Counts vs. Deconvoluted Mass (amu)

Figure S10: JW44_1708 active site mutant testing to probe presence of second active site. (A) Alphafold predicted structures highlighting the canonical catalytic triad and polymer binding groove (left, dark green) and the putative second catalytic triad and polymer binding groove (right, cyan). (B) Emulsified PLA turbidimetric assay against active site mutants. WT JW44_1708 and S77A and S120A active site mutants were purified small scale as described in the Materials and Methods and 0.05 mg/mL of each purified enzyme was incubated with 20% v/v of a low-molecular weight PLA (Mw ~ 10,000) emulsion in 50 mM Tris-HCl buffer for 2 hours at 37 °C and varying pH from 6.5 - 9.5. Traces represent averages of triplicate experiments. Precipitation was observed in the S77A reactions at pH values from 6.5 – 8.0, which disrupted the PLA emulsion accounting for the large signal fluctuations observed. Precipitation was not observed when the S77A mutant was incubated in buffer alone or for the PLA emulsion without enzyme. (C) The turbidimetric assay post 2 hours of reaction at pH 6.5 and pH 9.5 for WT JW44_1708 in Figure B was analyzed by normal-phase HPLC. A lactic acid peak was only observed at pH 9.5 and not at pH 6.5 (D) Mass spectrometry analysis of PMSF inhibited JW44_1708. Purified JW44_1708 was incubated for 30 mins at room temperature in phosphate buffer alone or with 1 mM PMSF and then analyzed by intact protein mass spectrometry. In the presence of PMSF there is a peak shift indicating addition of a single PMSF molecule. The second minor peak in the JW44_1708 + PMSF trace corresponds to unmodified JW44_1708 enzyme. Lack of double addition of PMSF, is suggestive of a single active site in JW44_1708.

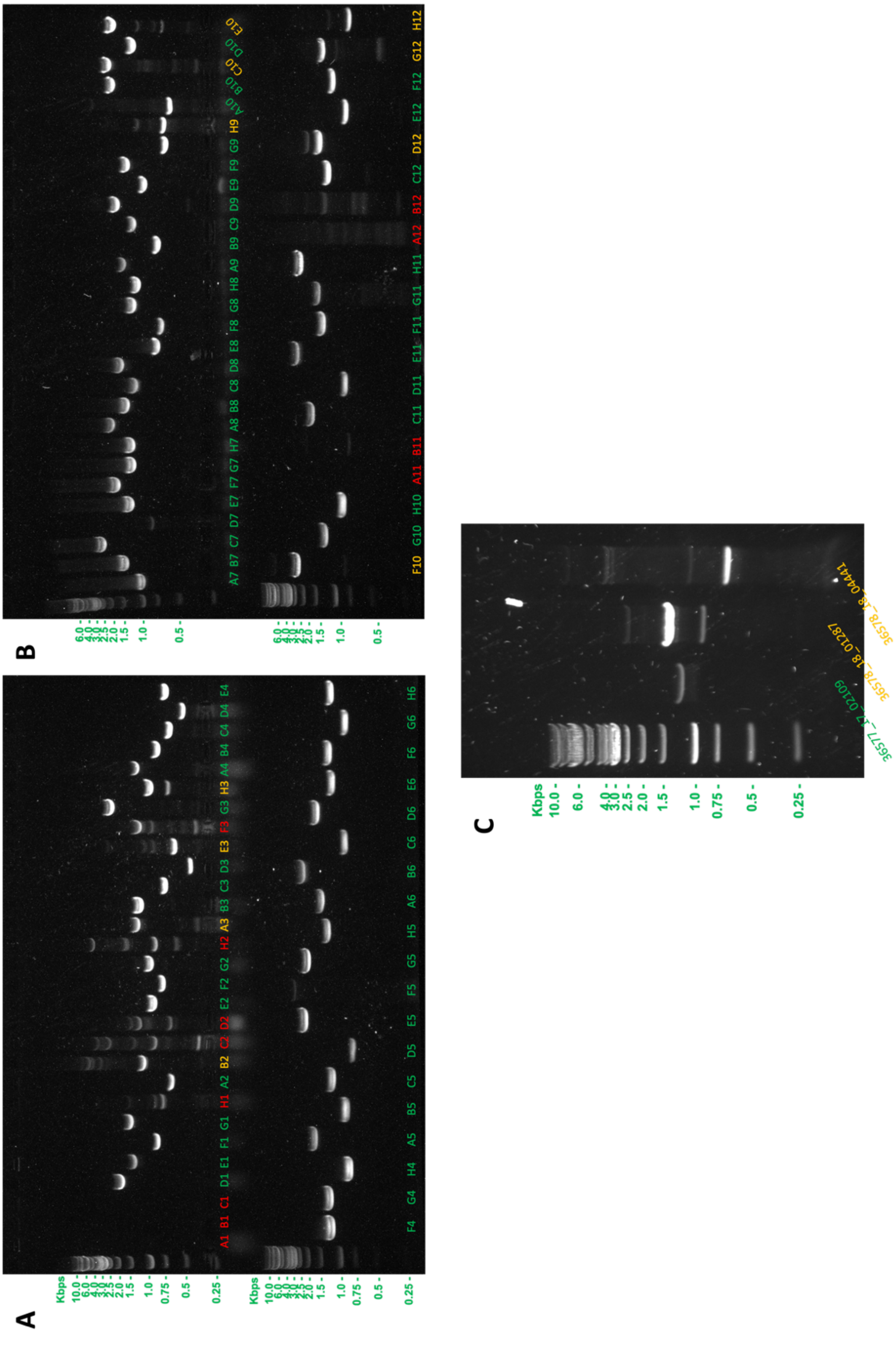

Figure S11: First pass PCR amplification of putative PDEs. 99 genes are shown. For clarity, gene products are identified by well location in figures A and B, see Table S6 for associated gene products in each well. Green denotes bands which amplified as single amplicons. Yellow denotes bands which were purified by gel extraction. Red denotes bands which did not amplify (either non-specific amplification or no product) on the first pass PCR.

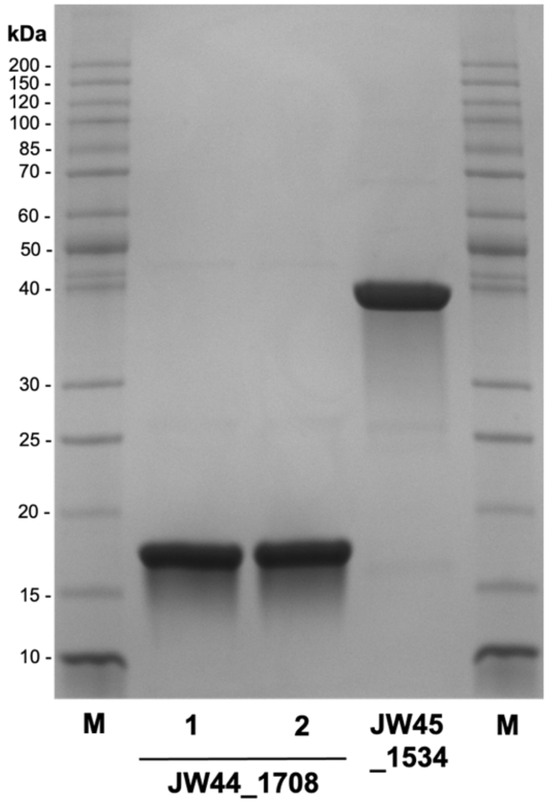

Figure S12: SDS-PAGE analysis of purified JW44_1708 and JW45_1534. JW44_1708 was run in two wells. M = protein ladder.

Supplementary Tables

Table S1: Source and locations of soil compost samples used in this study.

Soil compost sample	Description	Date of Sampling	Location
Home compost	Compost obtained from a domestic compost bin (composted for 6 months)	June 2019	Welwyn Garden City, United Kingdom
Wood chips	Bulking agent used for a commercial home hotbin	June 2021	Department of Biochemical Engineering, UCL
Industrial compost	Finished, screened compost taken from a PAS100 accredited commercial composting plant	April 2019	Commercial composting plant
Industrial leachate	Concentrated leachate from a PAS100 accredited commercial composting plant	December 2019	Commercial composting plant
Teabag compost	Compost obtained from a domestic compost bin co-fed with food waste and teabags	June 2021	Chislehurst, United Kingdom
PET hotbin	Sample from a commercial hotbin fed for 12 months with PET plastic	September 2021	Gordon Square, London, United Kingdom
Nylon/lycra hotbin	Sample from a commercial hotbin fed for 6 months with Nylon plastic	March 2021	Gordon Square, London, United Kingdom

Table S2: Genome sequencing statistics and soil of isolation

Strain	Contigs	bp	CDS	tRNA	tmRNA	CRISPR	Organism of highest identity[a]	Soil of isolation
JW1738	171	4589939	4425	90	1	-	*Lysinibacillus sphaericus (49.41%)*	Hotbin starter
JW1739	1446	5548841	5169	74	1	-	*Ochrobactrum anthropi (60.58%)*	Teabag compost
JW1740	272	3699882	3480	53	1	-	*Hydrogenophaga sp. (5.33%)*	Teabag compost
JW1741	122	3507954	3501	113	1	2	*Aneurinibacillus sp. (92.19%)*	Hotbin starter
JW1742	422	3992356	3852	85	1	8	*Aeribacillus pallidus (80.58%)*	Industrial compost
JW1743	278	5293778	5036	123	1	2	*Brevibacillus agri (4.69%)*	Industrial compost
JW1744	69	4292502	4392	80	1	-	*Bacillus licheniformis (32.10%)*	Teabag compost
JW1745	283	4059955	3733	74	0	5	*Bacillus thermoamylovorans (73.67%)*	Industrial leachate
JW1746	213	5674676	5339	132	1	3	*Brevibacillus agri (87.02%)*	Industrial compost
JW1747	212	5605589	5311	133	1	3	*Brevibacillus agri (88.20%)*	Industrial leachate
JW1748	79	4704386	4678	83	1	-	*Lysinibacillus sp. (43.87%)*	Teabag compost
JW1749	222	5608220	5307	133	1	3	*Brevibacillus agri (87.40%)*	Industrial leachate
JW1750	183	3095161	2815	48	1	4	*Bordetella avium (3.44%)*	Hotbin starter
JW1751	180	5878589	5974	98	1	-	*Bacillus cereus (65.08%)*	Industrial compost
JW1752	112	4074376	3928	118	1	-	*Rummeliibacillus stabekisii (5.21%)*	Industrial leachate
JW1753	240	5609029	5311	133	1	3	*Brevibacillus agri (87.27%)*	Industrial leachate
JW1754	177	5759698	5828	82	1	-	*Bacillus cereus (67.38%)*	Industrial compost
JW1755	245	5679013	5339	129	1	3	*Brevibacillus agri (86.36%)*	Industrial compost
JW1756	159	5649372	5804	100	1	-	*Bacillus cereus (49.03%)*	Industrial compost
JW1757	372	5839308	5529	136	1	3	*Brevibacillus agri (83.48%)*	Teabag compost

JW1758	1417	7467479	7379	121	2	5	*Bacillus licheniformis (28.40%)*	Industrial compost
JW1759	120	4205857	4163	81	1	-	*Bacillus paralicheniformis (92.86%)*	PET hot bin
JW1760	698	5034737	5230	93	1	-	*Bacillus licheniformis (33.60%)*	Impranil emulsion plate

[a] = Organism of highest identity was determined using Kraken.

Table S3: Signal peptide prediction statistics

Strain	OTHER	SP	TAT	LIPO	TATLIPO	PILIN	TOTAL	Total sequences with signal peptide predictions	% with signal peptides
JW1738	4118	162	139	3	0	3	4425	307	7
JW1739	4625	419	72	45	4	4	5169	544	11
JW1740	3108	208	73	70	9	12	3480	372	11
JW1741	3303	103	5	84	0	6	3501	198	6
JW1742	3693	59	2	95	0	3	3852	159	4
JW1743	4599	225	7	200	0	5	5036	437	9
JW1744	4109	166	3	112	0	2	4392	283	6
JW1745	3565	65	2	95	0	6	3733	168	5
JW1746	4896	229	4	204	0	6	5339	443	8
JW1747	4872	219	4	210	0	6	5311	439	8
JW1748	4389	139	3	144	0	3	4678	289	6
JW1749	4865	222	4	210	0	6	5307	442	8
JW1750	2500	211	27	69	2	6	2815	315	11
JW1751	5541	240	1	189	0	3	5974	433	7
JW1752	3671	137	3	112	0	5	3928	257	7
JW1753	4873	218	4	210	0	6	5311	438	8
JW1754	5401	235	1	188	0	3	5828	427	7
JW1755	4895	230	4	204	0	6	5339	444	8
JW1756	5395	210	2	194	0	3	5804	409	7
JW1757	5066	241	4	212	0	6	5529	463	8
JW1758	6962	232	6	174	0	5	7379	417	6
JW1759	3890	159	3	109	0	2	4163	273	7
JW1760	4939	170	3	116	0	2	5230	291	6

Table S4: Clan annotations of previously characterised PDEs from the PlasticDB. Clans which contain enzymes which degrade PLA/PBAT are highlighted in bold.

Clan	Number of occurrences
**CL0028**	**109**
CL0159	13
CL0592	9
**CL0013**	**8**
CL0635	4
CL0026	2
CL0063	2
**CL0264**	**2**
CL0713	2
CL0083	2
**CL0570**	**2**
CL0099	2
CL0186	1
CL0090	1
CL0502	1
CL0029	1
**CL0124**	**1**
CL0037	1

Table S5: Known PDE Pfam Family-specific functional annotations within clans.

Clan	Pfam family functional annotation
CL0013	Beta-lactamase, Beta-lactamase2
CL0028	DLH, Esterase_PHB, PAF-AH_p_II, Peptidase_S9, Hydrolase_4, Chlorophyllase2, Abhydrolase_1, Abhydrolase_6, Cutinase, Chlorophyllase, Esterase, BD-FAE, Abhydrolase_3, Abhydrolase_5, Lipase_2, PHB_depo_C, Coesterase, PE-PPE, Abhydrolase_2, FSH1, 3HBOH, Palm_thioest, DUF676, Ndr, Tannase, Say1_Mug180, DUF3141, DUF915, PGAP1, LCAT
CL0124	Trypsin, Trypsin2
CL0264	Lipase_GDSL, Lipase_GDSL_2, Lipase_GDSL_3
CL0570	Inhibitor_I9

Table S6: Table of enzymes tested for functional screening.

Sequence identifier	Strain of origin	Soil of origin	% GC	length (bp)	length (aa)	MW (Da)	Well location[a]
36577_17_01634	JW1738	Hotbin starter	40	1053	351	38895	A1
36577_17_01774	JW1738	Hotbin starter	37	876	292	33335	B1
36577_17_02109	JW1738	Hotbin starter	41	1245	415	44835	-
36577_17_03078	JW1738	Hotbin starter	36	2190	730	82319	C1
36577_17_03480	JW1738	Hotbin starter	41	2004	668	73348	D1
36577_17_03964	JW1738	Hotbin starter	36	1413	471	52492	E1
36577_17_04175	JW1738	Hotbin starter	40	861	287	31415	F1
36577_17_04346	JW1738	Hotbin starter	38	1539	513	55746	G1
36578_18_00298	JW1739	Teabag compost	59	750	250	27431	H1
36578_18_00417	JW1739	Teabag compost	59	627	209	22427	A2
36578_18_00677	JW1739	Teabag compost	59	1188	396	43184	B2
36578_18_00973	JW1739	Teabag compost	62	1440	480	49941	D12
36578_18_01287	JW1739	Teabag compost	57	1452	484	50961	-
36578_18_01403	JW1739	Teabag compost	56	996	332	35268	C2
36578_18_01466	JW1739	Teabag compost	59	1350	450	47299	D2
36578_18_01935	JW1739	Teabag compost	62	1131	377	40016	A12
36578_18_02199	JW1739	Teabag compost	58	1017	339	34471	E2
36578_18_02786	JW1739	Teabag compost	52	819	273	30271	F2
36578_18_03663	JW1739	Teabag compost	58	1083	361	39470	F12

36578_18_03789	JW1739	Teabag compost	57	1098	366	39603	G2
36578_18_04052	JW1739	Teabag compost	62	1284	428	43959	B12
36578_18_04219	JW1739	Teabag compost	60	807	269	29036	E12
36578_18_04437	JW1739	Teabag compost	58	948	316	34513	H2
36578_18_04440	JW1739	Teabag compost	60	1221	407	45653	C12
36578_18_04441	JW1739	Teabag compost	58	711	237	25122	-
36588_28_00083	JW1740	Teabag compost	70	573	191	20358	-
36588_28_00912	JW1740	Teabag compost	71	852	284	30907	-
36588_28_00931	JW1740	Teabag compost	69	738	246	26089	-
36588_28_01126	JW1740	Teabag compost	72	735	245	24792	-
36588_28_02458	JW1740	Teabag compost	69	1371	457	48425	-
36588_28_02917	JW1740	Teabag compost	69	1752	584	63888	-
235411_JW1741_01246	JW1741	Hotbin starter	49	1356	452	49776	B3
235412_JW1742_01051	JW1742	Industrial compost	39	774	258	29026	C3
235412_JW1742_02378	JW1742	Industrial compost	38	417	139	15304	D3
235412_JW1742_02532	JW1742	Industrial compost	42	2172	724	79499	B10
235413_JW1743_00008	JW1743	Industrial compost	51	612	204	22267	E3
235413_JW1743_01387	JW1743	Industrial compost	52	2466	822	90708	C10
235413_JW1743_01581	JW1743	Industrial compost	46	1371	457	52037	D10
235413_JW1743_01931	JW1743	Industrial compost	52	2166	722	79127	E10
235413_JW1743_02098	JW1743	Industrial compost	55	1386	462	50211	F3

235413_JW1743_03329	JW1743	Industrial compost	47	2562	854	96722	G3
235414_JW1744_00202	JW1744	Teabag compost	50	1053	351	35648	H3
235414_JW1744_00731	JW1744	Teabag compost	48	1428	476	51336	A4
235414_JW1744_01041	JW1744	Teabag compost	31	912	304	33359	B4
235414_JW1744_01500	JW1744	Teabag compost	45	699	233	26460	C4
235414_JW1744_01708	JW1744	Teabag compost	45	525	175	18491	D4
235414_JW1744_02003	JW1744	Teabag compost	46	771	257	28353	E4
235414_JW1744_02676	JW1744	Teabag compost	47	1260	420	45873	F4
235414_JW1744_03450	JW1744	Teabag compost	48	1401	467	51407	A3
235414_JW1744_03566	JW1744	Teabag compost	46	1284	428	47961	G4
235414_JW1744_03654	JW1744	Teabag compost	44	846	282	31173	H4
235414_JW1744_03710	JW1744	Teabag compost	49	1692	564	62136	A5
235414_JW1744_03712	JW1744	Teabag compost	49	861	287	30472	B5
235430_JW1745_01534	JW1745	Industrial leachate	39	1158	386	43447	C5
235430_JW1745_02547	JW1745	Industrial leachate	36	738	246	27637	D5
235430_JW1745_03477	JW1745	Industrial leachate	39	2160	720	79382	E5
235415_JW1746_00356	JW1746	Industrial compost	60	684	228	24718	H9
235415_JW1746_01730	JW1746	Industrial compost	52	2745	915	104133	F5
235415_JW1746_01871	JW1746	Industrial compost	56	2487	829	89477	F10
235416_JW1747_00763	JW1747	Industrial leachate	34	1269	423	46865	G10

235417_JW1748_00325	JW1748	Teabag compost	41	2013	671	73613	G5
235417_JW1748_00674	JW1748	Teabag compost	40	876	292	32970	H10
235417_JW1748_02109	JW1748	Teabag compost	41	1236	412	44498	H5
235417_JW1748_02598	JW1748	Teabag compost	35	1404	468	52455	A6
235417_JW1748_03056	JW1748	Teabag compost	36	2184	728	81768	B6
235417_JW1748_04017	JW1748	Teabag compost	40	1359	453	50377	A11
235417_JW1748_04264	JW1748	Teabag compost	37	831	277	30549	C6
235417_JW1748_04447	JW1748	Teabag compost	38	1542	514	55884	D6
235419_JW1750_00651	JW1750	Hotbin starter	64	597	199	21289	A10
235419_JW1750_00833	JW1750	Hotbin starter	65	1371	457	48255	G12
235419_JW1750_01395	JW1750	Hotbin starter	64	759	253	27556	H12
235420_JW1751_00168	JW1751	Industrial compost	38	1077	359	39126	E6
235420_JW1751_00566	JW1751	Industrial compost	36	1164	388	43189	F6
235420_JW1751_00606	JW1751	Industrial compost	34	2595	865	94887	B11
235420_JW1751_00636	JW1751	Industrial compost	38	816	272	29926	G6
235420_JW1751_00852	JW1751	Industrial compost	37	1110	370	39256	H6
235420_JW1751_00952	JW1751	Industrial compost	40	1062	354	38395	A7
235420_JW1751_01026	JW1751	Industrial compost	36	1521	507	55927	B7
235420_JW1751_01223	JW1751	Industrial compost	33	1755	585	64789	C11
235420_JW1751_01821	JW1751	Industrial compost	35	828	276	31278	D11

235420_JW1751_01829	JW1751	Industrial compost	37	2679	893	96533	C7
235420_JW1751_03923	JW1751	Industrial compost	39	816	272	30970	D7
235420_JW1751_04015	JW1751	Industrial compost	35	1398	466	52209	E7
235420_JW1751_04262	JW1751	Industrial compost	37	1929	643	70505	F7
235420_JW1751_05824	JW1751	Industrial compost	34	1338	446	47975	G7
235421_JW1752_00837	JW1752	Industrial leachate	40	1380	460	49361	H7
235421_JW1752_01299	JW1752	Industrial leachate	33	2154	718	78683	A8
235421_JW1752_02844	JW1752	Industrial leachate	39	1539	513	54674	B8
235421_JW1752_03333	JW1752	Industrial leachate	31	1305	435	47886	C8
235421_JW1752_03434	JW1752	Industrial leachate	33	1845	615	67075	D8
235421_JW1752_03630	JW1752	Industrial leachate	46	843	281	30838	E8
235421_JW1752_03876	JW1752	Industrial leachate	34	765	255	28950	F8
235421_JW1752_03906	JW1752	Industrial leachate	39	1395	465	50353	G8
235425_JW1756_00777	JW1756	Industrial compost	35	1284	428	46210	H8
235425_JW1756_01417	JW1756	Industrial compost	37	1710	570	63448	A9
235425_JW1756_01702	JW1756	Industrial compost	39	816	272	29717	B9
235425_JW1756_01760	JW1756	Industrial compost	35	2589	863	94496	E11
235425_JW1756_01767	JW1756	Industrial compost	31	1365	455	50901	C9
235425_JW1756_02726	JW1756	Industrial compost	41	2001	667	73427	D9
235425_JW1756_04631	JW1756	Industrial compost	38	1077	359	39021	E9

235425_JW1756_04867	JW1756	Industrial compost	39	1377	459	51022	F11
235426_JW1757_00335	JW1757	Teabag compost	52	1509	503	54599	G11
235428_JW1759_00548	JW1759	PET hot bin	49	1539	513	56746	F9
235428_JW1759_01525	JW1759	PET hot bin	45	681	227	25678	G9
235428_JW1759_02163	JW1759	PET hot bin	49	2337	779	82947	H11

[a]= Well location is provided for reference with Figure S11.

Table S7: Automated molecular biology pipeline success rates.

Pipeline step	Success rate (%)[a]
Automated PCR	75 (84)[b]
Automated cloning + transformation	90[c]
Expression strain transformation	99

[a] = Success rate is defined as the number of genes obtained for each of the cloning steps as a percentage of total genes processed on the first pass (i.e. without repeating/optimising steps).
[b] = 75% of genes gave a single band after PCR, the number in brackets accounts for the additional 9% of genes which were amplified cleanly for gel purification.
[c] = The percentage of genes which were successfully cloned on the first pass.

Table S8: Expression conditions optimisation.

Conditions – 37 °C/3 hrs, 18 hrs/25 °C/1000 rpm

Culture volume	0.25 mL	0.5 mL	1 mL
**Optical density**	2.756 ± 0.553	1.847 ± 0.287	1.05 ± 0.294

Conditions – 37 °C/3 hrs, 24 hrs/25 °C/0.5 mL culture volume

Shaking speed	1000 rpm	1250 rpm	1500 rpm
**Optical density**	1.863 ± 0.246	1.879 ± 0.186	2.778 ± 0.624

Conditions – 37 °C/3 hrs, 25 °C/0.5 mL culture volume/1500 rpm

Expression time	18 hrs	21 hrs	24 hrs
**Optical density**	2.016 ± 0.287	2.326 ± 0.402	2.778 ± 0.624

Conditions – 37 °C/3 hrs, 25 °C/0.25 mL culture volume/1800 rpm + breathable seal

Expression time	20 hrs
Optical density	10.724 ± 2.210

Table S9: Table of potential PLA degrading enzymes identified in preliminary functional screen.

Well	Enzyme	Pfam annotations	PLAase_ HMM hit	PBATase_HMM hit
A10	JW1739_01935	DUF900	+	-
A12	JW1748_00674	MIF4G_like_2, dsDNA_bind, Lipase_GDSL_2, Tristanin_u2	-	-
B11	JW1738_02109		-	-
B4	JW1744_01041	Peptidase_S8, Peripla_BP_4, DUF3181, Phage_holin_2_1	+	-
B5	JW1744_03712	Trypsin	-	-
**B7**	**JW1751_01026**	**PGAP1, P_proprotein, Lipoprotein_16**	**-**	**-**
C1	JW1738_03078	Peptidase_S9	-	-
**C5**	**JW1745_01534**	**DUF676, Imm40**	**+**	**+**
C8	JW1752_03333	Trypsin_2, Trypsin	-	-
**D4**	**JW1744_01708**	**Lipase_GDSL_2**	**+**	**+**
D7	JW1751_03923	Abhydrolase_1, DUF4263, Abhydrolase_1	+	+
E2	JW1739_02199	Peptidase_S58	-	-
E7	JW1751_04015	Beta-lactamase, coiled-coil_56, TMEM65, DUF2207, DUF998	-	-
E8	JW1752_03630	Beta-lactamase, Beta_lactamase2	-	-
F3	JW1743_02098	Beta-lactamase, DUF1129, Yfh0, Glucos_trans_II, RCR, DUF2070, DUF3810	-	-

Enzymes in bold were confirmed to have PLA degrading activity in secondary screening.

Table S10: PCR primers.

Enzyme sequence	Forward primer	Reverse primer
36577_17_01774	AAAGCTCTTCGATGGCAATATCAATAGATGAACCGAGTCCG	AAAGCTCTTCGGTGCTCCCCGAAGCCTATTTCCT
36577_17_02109	AAAGGTCTCTTATGTCGCCAAAAGAAAATGCAACAC	AAAGGTCTCGGGTGCTGTTCATTGGCGATAAATTTGTGA
36577_17_03078	AAAGCTCTTCGATGAATGAAACGAGCAATGTAATTGTTGCACAAGAGG	AAAGCTCTTCGGTGTTTTTTTAAATGGCTGTCGAAGAATTTAACC
36577_17_03480	AAAGCTCTTCGATGTCTAACAGTCAGACTGCGGTTGTGGCTAG	AAAGCTCTTCGGTGGTTCAACGTGATTGCAAATTCCG
36577_17_03964	AAAGCTCTTCGATGAGCGATCCTGTCATGGATAAAGATAAAACC	AAAGCTCTTCGGTGATTATTTCCAGGTAACCTTGAAACGAATATAGG
36577_17_04175	AAAGCTCTTCGATGGGTGAAAATTATGTAGCACTTGGAGATTCCTTAGC	AAAGCTCTTCGGTGAATTCGTTGTGGCTGTATGGATTGATTGGCTTTGAC
36577_17_04346	AAAGCTCTTCGATGAAGAAAGAAGATCATATAGCGCCAAGC	AAAGCTCTTCGGTGTTTCAAACGTTCACTAAAGTTCACTAATATTTTAGC
36578_18_00298	AAAGCTCTTCGATGGCCATTCCCGCCTCG	AAAGCTCTTCGGTGGCTTTTCCGGTTCGCG
36578_18_00417	AAAGCTCTTCGATGGAAGAACCGACCGGCCTTG	AAAGCTCTTCGGTGGCCTCCCGATGTCCCCTTG
36578_18_00677	AAAGCTCTTCGATGGCCAGCCGCCCTGAAGG	AAAGCTCTTCGGTGCTGCGTCGGAATCGTCACC
**36578_18_00973**	**aactttaagaaggagatatacatATGGCCGTGCAGGTAACGCC**	**gtggtggtgatgatgatgctcgagTCCCTGATCGATCGGCAG**
36578_18_01287	AAAGGTCTCTTATGCAGGGGCCAGCTTCCG	AAAGGTCTCGGGTGATTGATCCGGACAGTCACAAAAC
36578_18_01403	AAAGCTCTTCGATGGAGGTCGGCCAGCGG	AAAGCTCTTCGGTGGGTTTTTGGCTGAGGCTGCAACG

36578_18_01466	AAAGCTCTTCGATGGATGCGCCCGCGGC	AAAGCTCTTCGGTGACGCACAAACTGGCGCAAC
36578_18_01935	AAAGCTCTTCGATGGACACGCCGCGCG	AAAGCTCTTCGGTGCGGACCAGCGGAGGC
36578_18_02199	AAAGCTCTTCGATGAACAAGGGAGGAGAAAGCGTGCTG	AAAGCTCTTCGGTGTTCCTTTGCGTAATCCTTGTGTGCGG
36578_18_02786	AAAGCTCTTCGATGGAGCCTGTCACTTCGTCGTTTGAC	AAAGCTCTTCGGTGGTTGTTTTTAACGATGGGGGCTTTTCC
**36578_18_03663**	**aactttaagaaggagatatacatATGGCCGGTAACAGGGCTGTCAC**	**gtggtggtgatgatgatgctcgagGCGGATAGCCAATCGGTC**
36578_18_03789	AAAGCTCTTCGATGGGCACTATAAATGATGAGGAACTTCGCC	AAAGCTCTTCGGTGCTCGCTTTTATCGATCGCCTG
36578_18_04052	AAAGCTCTTCGATGCCGGGCGTTGCGGG	AAAGCTCTTCGGTGTCTCACAACCAGCAGAAGGC
**36578_18_04219**	**aactttaagaaggagatatacatATGGGACAGCCCGTTTCGACC**	**gtggtggtgatgatgatgctcgagCTTCGACTTCATCGAGACCG**
36578_18_04437	AAAGCTCTTCGATGCCGGCCGGGGC	AAAGCTCTTCGGTGGAATTTGGCAACGTCCAAAATCG
36578_18_04440	AAAGCTCTTCGATGATTGCGAAGCCCGTGAGGGTAG	AAAGCTCTTCGGTGCCGGAGACCTTTGAACGCTGC
36578_18_04441	AAAGCTCTTCGATGGAGGACAAACCTACTGTCGTGCTGGTGC	AAAGCTCTTCGGTGCCTTGCAGCGGCAGCTTC
36588_28_00083	AAAGCTCTTCGATGGGCGTGCAGCCCGTC	AAAGCTCTTCGGTGGCGGGGCAGCAGC
36588_28_00912	AAAGCTCTTCGATGGCCGCCCTCGACGAG	AAAGCTCTTCGGTGGTGGCCGCCGCCGTG
36588_28_00931	AAAGCTCTTCGATGGGGCTGACGACGGGGG	AAAGCTCTTCGGTGGGCCACGCCATGCTGCTGG
36588_28_01126	AAAGCTCTTCGATGGTGGGTGGCGGTGGCGG	AAAGCTCTTCGGTGGAGGATCTCCACGCCGG

36588_28_02458	AAAGCTCTTCGATGACCGGGGCTCCCCTGG	AAAGCTCTTCGGTGCCGCGTGGGCCGG
36588_28_02917	AAAGCTCTTCGATGTCCAGCTCGGGTTCCG	AAAGCTCTTCGGTGGAAGCGCAGGCCGG
235411_JW1741_01246	AAAGCTCTTCGATGTTTCTTCTGGCCCAGCCAG	AAAGCTCTTCGGTGTGGAAACAATTTCTGAAGCTGTTCCCAG
235412_JW1742_01051	AAAGCTCTTCGATGGTCCAAAAATCCACTGCTTCTCCTTC	AAAGCTCTTCGGTGATCCTTTTTTGTTCCATTTTCCAATGTTTC
235412_JW1742_02378	AAAGCTCTTCGATGGAAGGAAACATTAATACAATCATTACTCCTTTTTC	AAAGCTCTTCGGTGGGCATCCACCCTCGC
235412_JW1742_02532	AAAGGTCTCTTATGACCTTCCCCGAACATCCAC	AAAGGTCTCGGGTGTTGACTTTTTAATATTTTTTTATCTAAAATGATTGTCG
235413_JW1743_00008	AAAGCTCTTCGATGGGAAGCCAGGAAACGGC	AAAGCTCTTCGGTGTTTTTGACGGTTCTTGATAAACTCCAGC
235413_JW1743_01387	AAAGGTCTCTTATGATTACCAATGACGAATATATCAACAATCAGAC	AAAGGTCTCGGGTGCAAACTGTAAGCCTTCGCCAG
235413_JW1743_01581	AAAGGTCTCTTATGACACCTAATCCTGATTCTCAGATCGG	AAAGGTCTCGGGTGCTCGTCTTTGAACAATTCAACAATCTC
235413_JW1743_01931	AAAGGTCTCTTATGCAACAAGGTGAGCGCAGG	AAAGGTCTCGGGTGTTCAGGATAGCTCATCGAGAGTTTATAGG
235413_JW1743_02098	AAAGCTCTTCGATGCAGCAGGCGATTTCCGC	AAAGCTCTTCGGTGGCCGTTCACTGCCATTTTCACTG
235413_JW1743_03329	AAAGCTCTTCGATGGAAAGGATGGGTCAACCCGTTGG	AAAGCTCTTCGGTGTGTTTCACGCTTTAAAGCGTCTTCC
235414_JW1744_00202	AAAGCTCTTCGATGCAGCCGGCGAAAAATGTTGAAAAG	AAAGCTCTTCGGTGTTGAGCGGCAGCTTCGAC
235414_JW1744_00731	AAAGCTCTTCGATGACAGGCACGGCAGCG	AAAGCTCTTCGGTGTTTAGTTTTTTTTGCTGCGTCAAAAGCT

235414_JW 1744_01041	AAAGCTCTTCGATGTCCGAGGAAGAATATTTAAA AAATCAATACAG	AAAGCTCTTCGGTGAAACTTTCTGTTACGGCTTTTTC GAATAAAG
235414_JW 1744_01500	AAAGCTCTTCGATGACGGAGTGGAATGCGGGTG TTGA	AAAGCTCTTCGGTGTTCTGCTGGGAGGTCTTCGTTT TTA
235414_JW 1744_01708	AAAGCTCTTCGATGTCCCACAATCCGGTCGTC	AAAGCTCTTCGGTGTTTCCCGCTGGCGGTC
235414_JW 1744_02003	AAAGCTCTTCGATGACTTCTAACAGGAATGCCGA AAATGTTC	AAAGCTCTTCGGTGTTTATTTTCCTTCTCTAAAAACG CCATAACC
235414_JW 1744_02676	AAAGCTCTTCGATGGAACAAGCAGGAGAACAGT ATCTTTTGCTTG	AAAGCTCTTCGGTGCTCCTTGTTCATCATTTTCAGCG C
**235414_JW 1744_03450**	**aactttaagaaggagatatacatATGGGATTTAAAGGAG GAGGCGGC**	**gtggtggtgatgatgatgctcgagATCGGACAAAGGGCTGTT TTC**
235414_JW 1744_03566	AAAGCTCTTCGATGACTGCCATCGGGAACGAGA C	AAAGCTCTTCGGTGTTGGTGATGTAACGCCTCGTAA ACAGCTG
235414_JW 1744_03654	AAAGCTCTTCGATGGGCAGCAATCACTCGAATG CC	AAAGCTCTTCGGTGTTTGCTTTCCATGTTTAAGGCTT TCACTACC
235414_JW 1744_03710	AAAGCTCTTCGATGTCCGGAAAAAACTATAAAAT CATCGG	AAAGCTCTTCGGTGTTCGCAGCCTCCGATC
235414_JW 1744_03712	AAAGCTCTTCGATGCCATCGCCTCATACTCCTGT TTC	AAAGCTCTTCGGTGTTGTGCGCTGTTTTTCCAGTTG GTC
235415_JW 1746_00356	AAAGCTCTTCGATGACAGCGATCTGGTATTTGCG GC	AAAGCTCTTCGGTGACGGGCAATGACATCGGTC
235415_JW 1746_01730	AAAGCTCTTCGATGGAGCGTGAGCTTTTCGTCG AAG	AAAGCTCTTCGGTGGTCTACTGAGTGCTTTTCTTGC GACAGGC
235415_JW 1746_01871	AAAGGTCTCTTATGGCGCCTGCTTCCAATGAC	AAAGGTCTCGGGTGCACTTGATACGATTTGGCGATT AATACA
235416_JW 1747_00763	AAAGGTCTCTTATGCAAAAACGGAATTCAGAGTT AAATGAATATATTTTATC	AAAGGTCTCGGGTGGTTTCCAATAAATATGTCATCAA TTTCAAAATGAT

235417_JW 1748_00325	AAAGCTCTTCGATGTCTAGTAGCAGTCAGACTACCATTGTAGCTAGTC	AAAGCTCTTCGGTGGTTCAGCGTGATGGTAAATTCCGAGTTAG
235417_JW 1748_00674	AAAGGTCTCTTATGGCAATATCAATCGATGAACCGAATC	AAAGGTCTCGGGTGCTCCTCGAAGCCTATTGCCTTG
235417_JW 1748_02109	AAAGCTCTTCGATGGGGCAAAAAGAGGCGACAG	AAAGCTCTTCGGTGCTTTTTCGTTTGTATAAATTGCTCTATCGCTGGTAG
235417_JW 1748_02598	AAAGCTCTTCGATGTCGAGGGATACCATTCATAAAATTGACC	AAAGCTCTTCGGTGGATATTCTTTTTAGTAACAGTGAGTGATAAACCTAATAATAAATATAGGGAGC
235417_JW 1748_03056	AAAGCTCTTCGATGGATGAAACGAACAGTGTCGTGGTAGCAC	AAAGCTCTTCGGTGTTTTAAGTGACTGTCGAAGAATTTCACCATAGC
235417_JW 1748_04017	AAAGGTCTCTTATGTCAGATTCGAAATCTGCGGC	AAAGGTCTCGGGTGTCGGTGAGTGTATACAAACCATGTAATG
235417_JW 1748_04264	AAAGCTCTTCGATGACAGAAAGCTATATAGCTATTGGAGATTCCTTAGCTGC	AAAGCTCTTCGGTGAATGTTTTCTATAAAAGCTTTATAGCCAGGAAATCC
235417_JW 1748_04447	AAAGCTCTTCGATGGAACAAAGTGATTACATAGCCCCGAGTTGG	AAAGCTCTTCGGTGTTTAATCAATACATGACTAAGGTTTGTAAATATTTTCGCTGC
235419_JW 1750_00651	AAAGCTCTTCGATGACCGGCTCAGAGGATCGG	AAAGCTCTTCGGTGTTCGAGCAGTGCTTCCAGATGC
235419_JW 1750_00833	AAAGCTCTTCGATGCCGGCTCCCGTGGC	AAAGCTCTTCGGTGTTTACGAGGCGTTATGGTCACCC
235419_JW 1750_01395	AAAGCTCTTCGATGACGGCCATTGAGACGGATGC	AAAGCTCTTCGGTGGGCCAGATACTTCGCGAACC
235420_JW 1751_00168	AAAGCTCTTCGATGTCTACTCAAAATATTTCTAATTCGTCACAAGC	AAAGCTCTTCGGTGTTTATTAAATTCAACTTGCATCATTTTATCAAACTGTGTAAG
235420_JW 1751_00566	AAAGCTCTTCGATGGAGAGTCAACAAAATAATTATCCGATCATTTTAG	AAAGCTCTTCGGTGTTTCGGTAAACGTGATAGCTTTTCAGC

235420_JW 1751_00606	AAAGGTCTCTTATGGAAACGGGCAAAATGCAAGT TG	AAAGGTCTCGGGTGTTTTTGTATCTTTCTAAATTTGC GAACTAAAGTGA
235420_JW 1751_00636	AAAGCTCTTCGATGGAAAAGACTGGACAAGTGA AACATAAAAATCAGG	AAAGCTCTTCGGTGTTTAATAGCATCGATTACAACTT CAGCC
235420_JW 1751_00852	AAAGCTCTTCGATGACATCATCTACTGATTACGT TCCTAACCAATTAATC	AAAGCTCTTCGGTGAGAAGCTTTATTTTCTTGTAATT GCTTCGCATATTGTAC
235420_JW 1751_00952	AAAGCTCTTCGATGCCGTCGCAAAATGTATCTAG TTCTTTAC	AAAGCTCTTCGGTGCTTGCTAAATTCAGCAAGTAAAA TACTTGTAAAAGGATC
235420_JW 1751_01026	AAAGCTCTTCGATGGTTGTAAAAGAGCTTAAAAC AGGCTTTCCAG	AAAGCTCTTCGGTGCTTCTCCACATAAACCGACTTAA CAATTGTAC
235420_JW 1751_01223	AAAGGTCTCTTATGTCTATAGAACAGCAGAGTAA ATTAGGAATAGAACGAG	AAAGGTCTCGGGTGTTTTATGTTCAATACATATGAAC CATCTTTATTCTCATATTTATAG
235420_JW 1751_01821	AAAGGTCTCTTATGAGTGCAGCAGAAAAGCCAG TAGAAC	AAAGGTCTCGGGTGTTTTTCAGTTATTATAGGGTTTT TGCCAAAG
235420_JW 1751_01829	AAAGCTCTTCGATGTCGCTGCAAAAGGAGAAAC AATTTAGTC	AAAGCTCTTCGGTGTTTCACATCTAAATCAACATTTA AATTAGATGTATTTCC
235420_JW 1751_03923	AAAGCTCTTCGATGAACTTCGTTGATAAGCCGAT TGCG	AAAGCTCTTCGGTGTTGTGGATGCTTCGCAAAGAAA GCTTGTAC
235420_JW 1751_04015	AAAGCTCTTCGATGTCGGGTACGTTGACTGATAG GAGTATAGAGG	AAAGCTCTTCGGTGTACGTTGTTTTTATTACTTTTAAT TTTCGGGAAAAATC
235420_JW 1751_04262	AAAGCTCTTCGATGTCACCAAAGCAAGGTCAAAA AATTG	AAAGCTCTTCGGTGGTTTTCGCTAATGGATGCATTTA CAAGCTC

235420_JW1751_05824	AAAGCTCTTCGATGAGTGGAAATGATCAACTATTTATGTTTACTGATTCAAATACTTCAG	AAAGCTCTTCGGTGTTTAATCAGTTCTAAAGCCTTATTAACATTTATTAGACC
235421_JW1752_00837	AAAGCTCTTCGATGCAGGAGCAGGAACATTATGTAATAGCGTAC	AAAGCTCTTCGGTGCTTACCCTTAGATTTATCATTCGATAAGAAATTATAAATATTC
235421_JW1752_01299	AAAGCTCTTCGATGGTTGCGACTGATCAAACTATAGATGTAATGATATCCTACA	AAAGCTCTTCGGTGATGAACTGTAATTTTCAAAGGCTTACTTTTAACCCC
235421_JW1752_02844	AAAGCTCTTCGATGACATCTAACCTTCAGACTTCAAAAGAGAAAGTG	AAAGCTCTTCGGTGATTATTAACTAAAACTTTTTTACTTTTTTCTTTTGTTTGTACTTGATTTGCTAGTGTACC
235421_JW1752_03333	AAAGCTCTTCGATGACGATGTGGGGAAAAATAGAATTTAAAAATGGAATG	AAAGCTCTTCGGTGTTTAAGAGCGATACGAATACCTCCAAATG
235421_JW1752_03434	AAAGCTCTTCGATGTCTTCAACAGGGACAGCATACAAAG	AAAGCTCTTCGGTGTTTCACTGTAAACTTTACTGCAAGTGAATTTTTGTATGCATTTC
235421_JW1752_03630	AAAGCTCTTCGATGTCGATGCCTACCCATAACTTTTCG	AAAGCTCTTCGGTGCTTTAGAGCGTTAAGTGCGACATTTGCAG
235421_JW1752_03876	AAAGCTCTTCGATGGAAGTACCAGATACCATGAGTAATTCACAAAAAG	AAAGCTCTTCGGTGTTCATTTGTGGATTCCTCCTTAAAATCG
235421_JW1752_03906	AAAGCTCTTCGATGTCCTCCCAAACTTCCCTTCC	AAAGCTCTTCGGTGTTTTTTTCGCTTATTGATAAGTTTCAATGATTGGTAGG
235425_JW1756_00777	AAAGCTCTTCGATGAATGACGATTCTAATAAAACACAAGTTCATTCTAATAAG	AAAGCTCTTCGGTGATCTGGAAAAACAATTAATCCCTCTTCTTTTTC
235425_JW1756_01417	AAAGCTCTTCGATGAATAAAGAAACAAACTACATAAATGAGAATGACGTGAAGTTAAATGG	AAAGCTCTTCGGTGTTTCACAATTGGCAATGCTACTTCACTTA

235425_JW 1756_01702	AAAGCTCTTCGATGGAAAAGACTGGACAAGTGAAACATAAAAATCAGG	AAAGCTCTTCGGTGCCTAAAAGCATTAACTATAACTTCAGCCGC
235425_JW 1756_01760	AAAGGTCTCTTATGGGAGCGGGTCAAATGCAAG	AAAGGTCTCGGGTGTATTTTTCTAAATTTGCGAACTAAAGTGAACACTATAC
235425_JW 1756_01767	AAAGCTCTTCGATGCAAAATATTAAAGTTACATTAGATAAGTATATTGAAAAATTTATAAAAGAGCAG	AAAGCTCTTCGGTGTACTTTTTTCCTTCTTAAGGCTATAAGAATTGATATTAGTCC
235425_JW 1756_02726	AAAGCTCTTCGATGTCTAGCAGTAAGACTCCGGTTGTATCTAGCC	AAAGCTCTTCGGTGGTTCAATGCGACTGTAAATTCGGAATTC
235425_JW 1756_04631	AAAGCTCTTCGATGTCTACTCAAAATATCTCTAATTTGTCAAAAGCGC	AAAGCTCTTCGGTGTTTATTAAATTCAATTTGCATCATTTTATTAAACTGCG
235425_JW 1756_04867	AAAGGTCTCTTATGTCAGATTCGAAATCTGTGGCAATAC	AAAGGTCTCGGGTGTTTTCGGACCGAGGCATC
235426_JW 1757_00335	AAAGGTCTCTTATGGCTCCTGCTCCGATGC	AAAGGTCTCGGGTGTTTCTCTAACTCACCATCGTCATC
235428_JW 1759_00548	AAAGCTCTTCGATGCATCACAGTCAGGATCTTTTTCTGTTC	AAAGCTCTTCGGTGTTTCTTGAGCGAATGTTTCAGCGG
235428_JW 1759_01525	AAAGCTCTTCGATGGGAGAGGACGATCCCAGAAAAG	AAAGCTCTTCGGTGAGAAGTTTTTATAACTTTACTTTCATTAATTCCTTCA
235428_JW 1759_02163	AAAGGTCTCTTATGTCCGTTCCCGATCAAAAGTC	AAAGGTCTCGGGTGTTCGACGATAAAAGGGTCTTCC
235430_JW 1745_01534	AAAGCTCTTCGATGACGAGAGGAAATGATTATCCGATTGTTCTC	AAAGCTCTTCGGTGTTTAGGTAACGAATGTACTCGGTTTGCTACATC
235430_JW 1745_02547	AAAGCTCTTCGATGTCTGTATCAACTCAACCGCTCAGG	AAAGCTCTTCGGTGTTCATCCCATTTTTTTACGCCT
235430_JW 1745_03477	AAAGCTCTTCGATGGACCAAAGTGATATTCAAATGCAATCACAAATG	AAAGCTCTTCGGTGTTCCAATTCTAGTCCACGTTCG

36577_17_01634	AAAGCTCTTCGATGGGCGATCAATCCGTTGCG	AAAGCTCTTCGGTGCTCCTCTGCTGTAAGGCGTATTAGTTTGTAATCG

Sequences highlighted in bold were amplified by CPEC cloning using overhangs specific to pET29a(+). All other sequences were cloned either using *Sap*I or *Bsa*I. All primers were designed to exclude the N-terminal secretion peptide as predicted by SignalP 6.0.

Table S11: PCR amplification conditions for 18 sequences which required optimisation.

Enzyme sequence	Well	PCR additives	Annealing Temperature [T_A] (°C)	Extension time [x] (seconds)
36577_17_01634	A1	10% DMSO	60	105
36577_17_01774	B1	No additive	60	60
36577_17_03078	C1	No additive	60	60
36578_18_00298	H1	10% DMSO	60	105
36578_18_01403	C2	10% DMSO + 1.5 M betaine	65	60
36578_18_01466	D2	10% DMSO + 1.5 M betaine	65	60
36578_18_04437	H2	10% DMSO	63	105
235413_JW1743_02098	F3	10% DMSO	63	105
235417_JW1748_04017	A11	10% DMSO	60	105
235420_JW1751_00606	B11	not amplified	not amplified	not amplified
36578_18_01935	-	10% DMSO + 1.5 M betaine	63	60
36578_18_04052	-	10% DMSO + 1.5 M betaine	63	60
36588_28_00083	-	10% DMSO + 1.5 M betaine	63	60
36588_28_00912	-	10% DMSO + 1.5 M betaine	63	60
36588_28_00931	-	10% DMSO	63	105
36588_28_01126	-	10% DMSO + 1.5 M betaine	63	60
36588_28_02458	-	10% DMSO + 1.5 M betaine	63	60
36588_28_02917	-	not amplified	not amplified	not amplified

Thermocycling conditions – 98 °C/5 min, 30 cycles of (98 °C/10 sec, T_A/30 sec, 72 °C/x), 72 °C/7 min.

Enzyme Sequences

Signal peptide sequences are underlined+bold, however all experimental results were obtained with proteins which excluded this signal peptide sequence.

JW44_1708

MRRHSFLSILLICMLSVVSVFSFRPSAASAASHNPVVMVHGIGGADYNFIGIKSYLQ
SQGWTSSELYAINFIDKTGNNINNAPRLSEYIKRVLNQTGASKVDIVAHSMGGANTLY
YIKNLDGADKVGHVVTLGGANRLVTNTAPQNDKISYTSIYSTSDYIVLNSLSKLDGAN
NVQISGVSHVGLLFSSKVNALIKDGLTASGK

JW45_1534

MKTLQKLFITAVFLFSFFAVSLSIQPEQQVRAETRGNDYPIVLVHGLGGWGKGELL
GYRYWGGLKDIEFYLNQTGHRTYVATVGPVSSNWDRAVELYYYIKGGTVDYGAAH
AKEHGHARFGRTYPGIYGQWDETNKIHLIGHSMGGQTSRMLVELLKSGSQKEQEY
YNEHPEEGISPLFTGGKNWVHSVTSLATPHNGSTFADQDQIVSFIKDFIIHLASAAGQ
KQESLIYDFKLDQWGLKRQPGESFHAYMNRVMTSPIWQSNDISAYDLTTFGAQELN
QWVKTYPDVYYLSYTGNATYRGVVTGNYYPIGTMHPLFTLISMQMGSYTRQSPAP
VIDRSWLPNDGIVNVVSAKYPFGHPNSPYDGTIKQGVWNSFPVMEGWDHMDFINF
VGSNTPGYFSIYGYYNDVANRVHSLPK

JW51_1026

MRLMRRCVALLIVFFIMVPMISTNVRAEVVKELKTGFPDQEVFTPGEWFLGQKPAN
YDESKPPILFVQGRNGNADSWYGKTVYHDMNDMYDYALKAGYQTVFIQLYDAAGK
GSASQWDNGKLLAQKLEEIYNHFGKKVNIVAHSKGGIDTQAALVGYGANRFVGNVI
TLATPHHGSNLADLSYSWWAGWLASILGQKDDGTYSLQMGEMAKFRSTIDNSPAA
KLNRYYTVTGTSWGPVFSALSMGGLYLSSYGSNDGLVNEWSAKLPYGTHLFTDSR
FDHDNIRKGSAVFSRIEPYLRTANVPVPALVASSTSSNENIEQLNPTSNQSILGGELP
QNQWIEQTVTVDKKAEGIVSVLTASSDVEVQVISPKGKMFTNKDSVITNDEGESFFG
GATIKTFKFDKMDVGEWKVKMMTKQSKDAYLIVSDYKSDAPFVLQMPTKVKANKSE
YKLKKSPVAPEMKGDLSITVRVVNKEGKLVSEFNELQNVNNNTFTGALKNIKQPGVY
NVTMDIKGMNKEGKSYSRTIVKSVYVEK

References

(1) Teufel, F.; Almagro Armenteros, J. J.; Johansen, A. R.; Gíslason, M. H.; Pihl, S. I.; Tsirigos, K. D.; Winther, O.; Brunak, S.; von Heijne, G.; Nielsen, H. SignalP 6.0 Predicts All Five Types of Signal Peptides Using Protein Language Models. *Nat Biotechnol* **2022**. https://doi.org/10.1038/s41587-021-01156-3.

(2) Dobrijevic, D.; Nematollahi, L. A.; Hailes, H. C.; Ward, J. M. PET Expression Vector Customized for Efficient Seamless Cloning. *Biotechniques* **2020**, *69* (5), 384–387. https://doi.org/10.2144/btn-2020-0101.

(3) Mirdita, M.; Schütze, K.; Moriwaki, Y.; Heo, L.; Ovchinnikov, S.; Steinegger, M. ColabFold: Making Protein Folding Accessible to All. *Nat Methods* **2022**, *19* (6), 679–682. https://doi.org/10.1038/s41592-022-01488-1.

(4) Hajighasemi, M.; Tchigvintsev, A.; Nocek, B.; Flick, R.; Popovic, A.; Hai, T.; Khusnutdinova, A. N.; Brown, G.; Xu, X.; Cui, H.; Anstett, J.; Chernikova, T. N.; Brüls, T.; Le Paslier, D.; Yakimov, M. M.; Joachimiak, A.; Golyshina, O. V.; Savchenko, A.; Golyshin, P. N.; Edwards, E. A.; Yakunin, A. F. Screening and Characterization of Novel Polyesterases from Environmental Metagenomes with High Hydrolytic Activity against Synthetic Polyesters. *Environ Sci Technol* **2018**, *52* (21), 12388–12401. https://doi.org/10.1021/acs.est.8b04252.

(5) Mayumi, D.; Akutsu-Shigeno, Y.; Uchiyama, H.; Nomura, N.; Nakajima-Kambe, T. Identification and Characterization of Novel Poly(DL-Lactic Acid) Depolymerases from Metagenome. *Appl Microbiol Biotechnol* **2008**, *79* (5), 743–750. https://doi.org/10.1007/s00253-008-1477-3.

(6) Akutsu-Shigeno, Y.; Teeraphatpornchai, T.; Teamtisong, K.; Nomura, N.; Uchiyama, H.; Nakahara, T.; Nakajima-Kambe, T. Cloning and Sequencing of a Poly(DL-Lactic Acid) Depolymerase Gene from Paenibacillus Amylolyticus Strain TB-13 and Its Functional Expression in Escherichia Coli. *Appl Environ Microbiol* **2003**, *69* (5), 2498–2504. https://doi.org/10.1128/AEM.69.5.2498-2504.2003.

(7) Hajighasemi, M.; Nocek, B. P.; Tchigvintsev, A.; Brown, G.; Flick, R.; Xu, X.; Cui, H.; Hai, T.; Joachimiak, A.; Golyshin, P. N.; Savchenko, A.; Edwards, E. A.; Yakunin, A. F. Biochemical and Structural Insights into Enzymatic Depolymerization of Polylactic Acid and Other Polyesters by Microbial Carboxylesterases. *Biomacromolecules* **2016**, *17* (6), 2027–2039. https://doi.org/10.1021/acs.biomac.6b00223.

(8) Tchigvintsev, A.; Tran, H.; Popovic, A.; Kovacic, F.; Brown, G.; Flick, R.; Hajighasemi, M.; Egorova, O.; Somody, J. C.; Tchigvintsev, D.; Khusnutdinova, A.; Chernikova, T. N.; Golyshina, O. V.; Yakimov, M. M.; Savchenko, A.; Golyshin, P. N.; Jaeger, K. E.; Yakunin, A. F. The Environment Shapes Microbial Enzymes: Five Cold-Active and Salt-Resistant Carboxylesterases from Marine Metagenomes. *Appl Microbiol Biotechnol* **2015**, *99* (5), 2165–2178. https://doi.org/10.1007/s00253-014-6038-3.

(9) Ribitsch, D.; Hromic, A.; Zitzenbacher, S.; Zartl, B.; Gamerith, C.; Pellis, A.; Jungbauer, A.; Łyskowski, A.; Steinkellner, G.; Gruber, K.; Tscheliessnig, R.; Herrero Acero, E.; Guebitz, G. M. Small Cause, Large Effect: Structural Characterization of Cutinases from Thermobifida Cellulosilytica. *Biotechnol Bioeng* **2017**, *114* (11), 2481–2488. https://doi.org/10.1002/bit.26372.

(10) Blázquez-sánchez, P.; Engelberger, F.; Cifuentes-anticevic, J.; Sonnendecker, C.; Griñén, A. Antarctic Polyester Hydrolases Degrade Aliphatic and Aromatic. *Appl Environ Microbiol* **2022**, *88* (1), 1–14.

(11) Kawai, F.; Oda, M.; Tamashiro, T.; Waku, T.; Tanaka, N.; Yamamoto, M.; Mizushima, H.; Miyakawa, T.; Tanokura, M. A Novel Ca2+-Activated, Thermostabilized Polyesterase Capable of Hydrolyzing Polyethylene Terephthalate from Saccharomonospora Viridis AHK190. *Appl Microbiol Biotechnol* **2014**, *98* (24), 10053–10064. https://doi.org/10.1007/s00253-014-5860-y.

(12) Kawai, F.; Nakadai, K.; Nishioka, E.; Nakajima, H.; Ohara, H.; Masaki, K.; Iefuji, H. Different Enantioselectivity of Two Types of Poly(Lactic Acid) Depolymerases toward

Poly(l-Lactic Acid) and Poly(d-Lactic Acid). *Polym Degrad Stab* **2011**, *96* (7), 1342–1348. https://doi.org/10.1016/j.polymdegradstab.2011.03.022.
(13) Oda, Y.; Yonetsu, A.; Urakami, T.; Tonomura, K. Degradation of Polylactide by Commercial Proteases. *J Polym Environ* **2000**, *8* (1), 29–32. https://doi.org/10.1023/A:1010120128048.
(14) Li, F.; Wang, S.; Liu, W.; Chen, G. Purification and Characterization of Poly(l-Lactic Acid)-Degrading Enzymes from Amycolatopsis Orientalis Ssp. Orientalis. *FEMS Microbiol Lett* **2008**, *282* (1), 52–58. https://doi.org/10.1111/j.1574-6968.2008.01109.x.
(15) Yamashita, K.; Kikkawa, Y.; Kurokawa, K.; Doi, Y. Enzymatic Degradation of Poly(L-Lactide) Film by Proteinase K: Quartz Crystal Microbalance and Atomic Force Microscopy Study. *Biomacromolecules* **2005**, *6* (2), 850–857. https://doi.org/10.1021/bm049395v.
(16) Mohanan, N.; Wong, M. C. H.; Budisa, N.; Levin, D. B. Polymer-Degrading Enzymes of Pseudomonas Chloroaphis PA23 Display Broad Substrate Preferences. *Int J Mol Sci* **2023**, *24* (5). https://doi.org/10.3390/ijms24054501.
(17) Jung, H. W.; Yang, M. K.; Su, R. C. Purification, Characterization, and Gene Cloning of an Aspergillus Fumigatus Polyhydroxybutyrate Depolymerase Used for Degradation of Polyhydroxybutyrate, Polyethylene Succinate, and Polybutylene Succinate. *Polym Degrad Stab* **2018**, *154*, 186–194. https://doi.org/10.1016/j.polymdegradstab.2018.06.002.
(18) Perz, V.; Baumschlager, A.; Bleymaier, K.; Zitzenbacher, S.; Hromic, A.; Steinkellner, G.; Pairitsch, A.; Łyskowski, A.; Gruber, K.; Sinkel, C.; Küper, U.; Ribitsch, D.; Guebitz, G. M. Hydrolysis of Synthetic Polyesters by Clostridium Botulinum Esterases. *Biotechnol Bioeng* **2016**, *113* (5), 1024–1034. https://doi.org/10.1002/bit.25874.
(19) Biundo, A.; Hromic, A.; Pavkov-Keller, T.; Gruber, K.; Quartinello, F.; Haernvall, K.; Perz, V.; Arrell, M. S.; Zinn, M.; Ribitsch, D.; Guebitz, G. M. Characterization of a Poly(Butylene Adipate-Co-Terephthalate)-Hydrolyzing Lipase from Pelosinus Fermentans. *Appl Microbiol Biotechnol* **2016**, *100* (4), 1753–1764. https://doi.org/10.1007/s00253-015-7031-1.
(20) Muroi, F.; Tachibana, Y.; Soulenthone, P.; Yamamoto, K.; Mizuno, T.; Sakurai, T.; Kobayashi, Y.; Kasuya, K. ichi. Characterization of a Poly(Butylene Adipate-Co-Terephthalate) Hydrolase from the Aerobic Mesophilic Bacterium Bacillus Pumilus. *Polym Degrad Stab* **2017**, *137*, 11–22. https://doi.org/10.1016/j.polymdegradstab.2017.01.006.
(21) Soulenthone, P.; Tachibana, Y.; Suzuki, M.; Mizuno, T.; Ohta, Y.; Kasuya, K. ichi. Characterization of a Poly(Butylene Adipate-Co-Terephthalate) Hydrolase from the Mesophilic Actinobacteria Rhodococcus Fascians. *Polym Degrad Stab* **2021**, *184*, 109481. https://doi.org/10.1016/j.polymdegradstab.2021.109481.
(22) Perz, V.; Hromic, A.; Baumschlager, A.; Steinkellner, G.; Pavkov-Keller, T.; Gruber, K.; Bleymaier, K.; Zitzenbacher, S.; Zankel, A.; Mayrhofer, C.; Sinkel, C.; Kueper, U.; Schlegel, K.; Ribitsch, D.; Guebitz, G. M. An Esterase from Anaerobic Clostridium Hathewayi Can Hydrolyze Aliphatic-Aromatic Polyesters. *Environ Sci Technol* **2016**, *50* (6), 2899–2907. https://doi.org/10.1021/acs.est.5b04346.
(23) Muller, C. A.; Perz, V.; Provasnek, C.; Quartinello, F.; Gübitz, G.; Berg, G. Discovery of Polyesterases from Moss-Associated Microorganisms. *Appl Environ Microbiol* **2017**, *83* (4), e02641-16.
(24) Fischer, E. W.; Sterzel, H. J.; Wegner, G. Investigation of the Structure of Solution Grown Crystals of Lactide Copolymers by Means of Chemical Reactions. *Kolloid-Zeitschrift Zeitschrift für Polymere* **1973**, *251*, 980–990.
